# Management of Rheumatoid Arthritis With a Digital Health Application: A Multicenter, Pragmatic Randomized Clinical Trial

**DOI:** 10.1001/jamanetworkopen.2023.8343

**Published:** 2023-04-14

**Authors:** Chun Li, Jianlin Huang, Huaxiang Wu, Fen Li, Yi Zhao, Zhenchun Zhang, Shengguang Li, Hua Wei, Miaojia Zhang, Hongsheng Sun, Jing Yang, Qin Li, Xiaomei Li, Wufang Qi, Wei Wei, Yasong Li, Zhenbin Li, Yongfu Wang, Fengxiao Zhang, Henglian Wu, Zongwen Shuai, Zhenbiao Wu, Yi Li, Shengsong Jia, Yuhua Jia, Fei Xiao, Rong Mu, Zhanguo Li

**Affiliations:** 1 Department of Rheumatology and Immunology, Peking University People’s Hospital, Beijing, China; 2 Department of Rheumatology, the Sixth Affiliated Hospital of Sun Yat-sen University, Guangzhou, Guangdong, China; 3 Department of Rheumatology and Immunology, the Second Affiliated Hospital of Zhejiang University School of Medicine, Hangzhou, Zhejiang, China; 4 Department of Rheumatology and Immunology, the Second Xiangya Hospital of Central South University, Changsha, Hunan, China; 5 Department of Rheumatology and Immunology, Xuanwu Hospital Capital Medical University, Beijing, China; 6 Department of Rheumatology and Immunology, Linyi People’s Hospital, Linyi, Shandong, China; 7 Department of Rheumatology and Immunology, Peking University International Hospital, Zhongguancun Life Science Park, Beijing, China; 8 Department of Rheumatology and Immunology, Northern Jiangsu People's Hospital, Yangzhou, Jiangsu, China; 9 Department of Rheumatology and Immunology, the First Affiliated Hospital of Nanjing Medical University, Nanjing, Jiangsu, China; 10 Department of Rheumatology and Immunology, Shandong Provincial Hospital Affiliated to Shandong First Medical University, Jinan, Shandong, China; 11 Department of Rheumatology and Immunology, Mianyang Central Hospital, Mianyang, Sichuan, China; 12 Department of Rheumatology and Immunology, the First People's Hospital of Yunnan Province, Kunming, Yunnan, China; 13 Department of Rheumatology and Immunology, the First Affiliated Hospital of USTC, Hefei, Anhui, China; 14 Department of Rheumatology and Immunology, Tianjin First Central Hospital, Tianjin, China; 15 Department of Rheumatology and Immunology, Tianjin Medical University General Hospital, Tianjin, China; 16 Department of Rheumatology and Immunology, Zhejiang Provincial People’s Hospital, Hangzhou, Zhejiang, China; 17 Department of Rheumatology and Immunology, Bethune International Peace Hospital, Shijiazhuang, Hebei, China; 18 Department of Rheumatology and Immunology, the First Affiliated Hospital of Baotou Medical College, Inner Mongolia University of Science and Technology, Baotou, Inner Mongolia, China; 19 Department of Rheumatology and Immunology, Hebei General Hospital, Shijiazhuang, Hebei, China.; 20 Department of Rheumatology and Immunology, Tungwah Hospital of Sun Yat-sen University, Dongguan, Guangdong, China; 21 Department of Rheumatology and Immunology, the First Affiliated Hospital of Anhui Medical University, Hefei, Anhui, China; 22 Department of Rheumatology and Immunology, Xijing Hospital, Xi'an, Shanxi, China; 23 School of Statistics and Mathematics, Nanjing Audit university, Nanjing, Jiangsu, China; 24 Shanghai Gothic Internet Technology Co, Shanghai, China; 25 Department of Rheumatology and Immunology, Peking University Third Hospital, Beijing, China; 26 State Key Laboratory of Natural and Biomimetic Drugs, School of Pharmaceutical Sciences, Peking University, Beijing, China.; 27 Peking-Tsinghua Center for Life Sciences, Peking University, Beijing, China

## Abstract

**IMPORTANCE:**

Digital health applications have been shown to be effective in the management of chronic diseases with simple treatment targets. The potential clinical value of digital health applications in rheumatoid arthritis (RA) has not been well studied.

**OBJECTIVE:**

To investigate whether assessing patient-reported outcomes using digital health applications could result in disease control for patients with RA.

**DESIGN, SETTING, AND PARTICIPANTS:**

This is a multicenter, open-label randomized clinical trial in 22 tertiary hospitals across China. Eligible participants were adult patients with RA. Participants were enrolled from November 1, 2018, to May 28, 2019, with a 12-month follow-up. The statisticians and rheumatologists who assessed disease activity were blinded. Investigators and participants were not blind to group assignment. Analysis was conducted from October 2020 to May 2022.

**INTERVENTIONS:**

Participants were randomly assigned at a 1:1 ratio (block size of 4) to a smart system of disease management group (SSDM) or a conventional care control group. Upon the completion of the 6-month parallel comparison, patients in the conventional care control group were instructed to use the SSDM application for an extension of 6 months.

**MAIN OUTCOMES AND MEASURES:**

The primary outcome was the rate of patients with disease activity score in 28 joints using the C-reactive protein (DAS28-CRP) of 3.2 or less at month 6.

**RESULTS:**

Of 3374 participants screened, 2204 were randomized, and 2197 patients with RA (mean [SD] age, 50.5 [12.4] years; 1812 [82.5%] female) were enrolled. The study included 1099 participants in the SSDM group and 1098 participants in the control group. At month 6, the rate of patients with DAS28-CRP of 3.2 or less was 71.0% (780 of 1099 patients) in the SSDM group vs 64.5% (708 of 1098 patients) in the control group (difference between groups, 6.6%; 95% CI, 2.7% to 10.4%; *P* = .001). At month 12, the rate of patients with DAS28-CRP of 3.2 or less in the control group increased to a level (77.7%) that was comparable with that (78,2%) in the SSDM group (difference between groups, −0.2%; 95% CI, −3.9% to 3.4%; *P* = .90).

**CONCLUSIONS AND RELEVANCE:**

In this randomized clinical trial of RA, the use of a digital health application with patient-reported outcomes was associated with an increase in disease control rate.

**TRIAL REGISTRATION:**

ClinicalTrials.gov Identifier: NCT03715595

## Key Points

**Question:**

What is the clinical value of a digital health application in the management of rheumatoid arthritis, a disease with complex treatment targets?

**Findings:**

In this randomized clinical trial of 2197 patients with rheumatoid arthritis, a statistically significant increase in the rate of DAS28-CRP of 3.2 or less at month 6 was observed with the use of a smartphone application for assessing patient-reported outcomes.

**Meaning:**

These findings suggest that assessing patient-reported outcomes using a smartphone application resulted in clinical improvement in disease activity for patients with rheumatoid arthritis.

## Teaser

This randomized clinical trial investigates whether using digital health applications to assess patient-reported outcomes could result in disease control in patients with rheumatoid arthritis at 22 tertiary hospitals in China.

## Introduction

Digital health applications are rapidly transforming the landscape of medical practice.^1,2,3,4^ For chronic diseases with clearly defined, simple treatment targets that can be monitored using biosensors, such as hypertension,^5^ digital health applications are particularly useful. In contrast, the use of digital health applications in diseases with more complex treatment targets, such as rheumatoid arthritis (RA), has not been proven.

Treat-to-target is the recommended strategy in the management of RA^6,7,8^ and requires standardized assessment that includes both objective and subjective evaluations. At the clinical level, treatment decision-making is not completely consistent with the treat-to-target approach,^9,10,11^ and failure to regularly assess disease activity using standardized tools remains a major obstacle.^11,12,13^ The 28-joint disease activity score (DAS28) is a commonly used tool for assessing disease activity in patients with RA.^14^ There is a need for the patient to participate in disease management not only in treatment decision-making but also in disease activity assessment.

Patient-reported outcomes (PROs) have been increasingly used in the management of chronic disease for a long history.^15,16,17,18,19,20,21,22,23^ PROs have not only been applied in determining the status and treatment of patients with RA, but are also being widely used in clinical trials. The core variables of PROs include patients’ self-assessment of disease activity, pain, and physical function.^20^ Additionally, other domains, including remission, flare, and self-management, are also reported.^24,25,26^ Furthermore, significant efforts have been made toward developing the digital health applications based on simplified PROs for patients with RA.^27,28,29,30^ However, in general, these tools only typically capture a snapshot of the disease spectrum. Two systematic reviews of digital applications for RA concluded that there was substantial room for improvement.^31,32^ Specifically, there has been a lack of tools that allow convenient, standardized, and comprehensive evaluation of disease activity by patients themselves. Lack of interaction between patients and physicians also needs to be improved. Two randomized clinical trials have been conducted to examine the efficacy of smartphone health applications in patients with RA.^30,33^ There was no statistically significant difference in the primary end point in either trial, but the reduction in rheumatologist consultations and positive experiences were confirmed.^30,33^^,^ Wearable devices in combination with smartphone health applications have also been developed in the management of RA.^34,35,36,37^ However, most wearable devices are not used for monitoring disease activity. It is important to evaluate PROs using a smartphone app in patients with RA.

We conducted a multicenter, open-label randomized clinical trial to compare SSDM with conventional care in patients with RA. The primary end point was the rate of patients with DAS28-CRP of 3.2 or less at month 6.

## Methods

### Study Design and Participants

**Figure 1.**
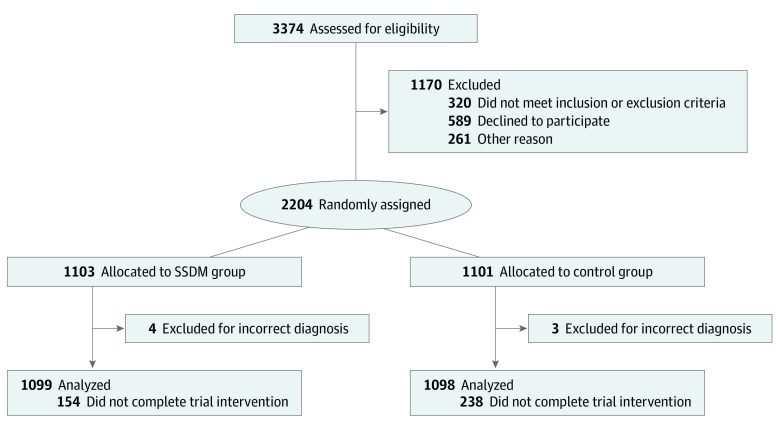
Trial Profile SSDM indicates Smart System of Disease Management.

This randomized clinical trial followed the Consolidated Standards of Reporting Trials (CONSORT) reporting guideline. The study protocol is provided in Supplement 1, and the CONSORT flow diagram is provided in Figure 1. This study was approved and monitored by the ethics committee of Peking University People’s Hospital. The investigators at each center screened potentially eligible participants, explained the trial to them, checked inclusion and exclusion criteria, and obtained written informed consent from all participants prior to their enrollment (eMethods in Supplement 2).

This randomized clinical trial was conducted at 22 tertiary hospitals across China. Adult patients aged 18 years or older who met the 2010 American College of Rheumatology (ACR)/European League Against Rheumatism (EULAR) criteria for RA were eligible.^38^ The completed inclusion and exclusion criteria are included in eTable 1 in Supplement 2. The trial consisted of a 6-month initial phase that compared SSDM management and conventional care, and a 6-month extension phase during which participants in both groups were invited to use SSDM management.

### Randomization and Masking

From November 1, 2018, to May 28, 2019, eligible patients were randomly assigned at a 1:1 ratio into a SSDM group vs a conventional care control group. All patients were followed up with for 12 months. The randomization sequence was generated with an interactive web response system using a block design (block sizes of 4). The randomization was stratified based on the DAS28-CRP score at baseline (ie, remission [REM], DAS28-CRP ≤ 2.6 and low disease activity [LDA], 2.6-3.2; moderate disease activity [MDA], 3.2-5.1; or high disease activity [HDA], > 5.1), as assessed by the rheumatologists. The statisticians and the rheumatologists who assessed DAS28-CRP were blinded to group allocation. Investigators and participants were not blind to group assignment.

### Intervention

Patients randomized to the SSDM group were asked to conduct self-assessment and report the results once every month by themselves. Patients randomized to the control group received conventional care and maintained their routine medical visits during the first 6 months and were asked to come back for a visit at month 6 and month 12.

Upon the first use of the system, a research staff was onsite to assist the patients with the following information: full name, sex, date of birth, date of initial diagnosis, comorbidities, education level, occupation, family income, annual medical expenses, and DAS28-CRP at each research site. Other information included: (1) laboratory results (eg, routine blood test, liver and kidney function, and CRP), submitted as photographs and automatically processed to extract key information via Optical Character Recognition technology; (2) medications, for RA as well as comorbid conditions; and (3) perceived adverse reactions (a total of 33 types of symptoms). The DAS28-CRP score, as assessed by patients, together with key laboratory reports if available, were uploaded and synchronized to a rheumatologist’s interface, and the assigned rheumatologists could monitor the patient’s condition online, as well as instruct the patients to come back for outpatient visits or refill or make new prescriptions.

The alert function of the SSDM was performed at 4 months after the trial started. A red flag was raised for 1 or more of the following conditions: (1) disease activity exacerbation—the DAS28-CRP score increased to and remained MDA for 3 months or increased to HDA in patients with REM or LDA at baseline; (2) sustained MDA or worsening HDA—the DAS28-CRP score remained at 3.2 to 5.1 for 3 months or increased to more than 5.1 in patients with MDA at baseline; (3) HDA status—the DAS28-CRP score remained higher than 5.1 for 3 months, decreased to between 3.2 and 5.1 but had subsequent exacerbation (ie, DAS28-CRP increased to >5.1) at any time point, or no further reduction by at least 1.2 within 3 months in patients with HDA at baseline. The alert was also triggered upon elevated alanine aminotransferase or aspartate aminotransferase levels above 2 times the upper normal limit or a white blood cell count of less than 2000 or greater than 10 000 per mL.

Patients in the SSDM group watched a 15-minute video that described the key features of the SSDM to allow them to correctly use of the application. The use of SSDM and self-assessment of DAS28-CRP by patients were confirmed by physicians.

### Outcomes

The primary outcome was the rate of patients with a DAS28-CRP of 3.2 or less at month 6, as assessed by a rheumatologist. Secondary outcomes were also evaluated by the rheumatologist, and included the proportion of patients with moderate-to-good EULAR response rate,^39^ ACR/EULAR Boolean remission rate,^40^ the change in simplified disease activity index (SDAI),^41^ the change in clinical disease activity index (CDAI),^42^ the change in tender joint count and swollen joint count, the change in Hospital Anxiety and Depression Scale,^43^ the change in the 36-Item Short Form Survey,^44^ the flare rate at month 6 and month 12, and the rate of patients with a DAS28-CRP of 3.2 or less at month 12. The numbers and rates of adverse events, either reported by the rheumatologists or resulting from an alert in the SSDM, were also compared. A flare was defined as an increase in a DAS28-CRP of more than 1.2 or more than 0.6 of the final DAS28-CRP of 3.2 or higher among patients with a DAS28-CRP of 3.2 or less at baseline.^45^ Adherence was defined as the ratio of actual self-assessment numbers against the required self-assessment numbers.

### Statistical Analysis

A sample size of 2200 patients was calculated to provide 90% power to detect a difference between the SSDM group and the control group at a 2-sided α level of .05, assuming that the 6-month rate of patients with a DAS28-CRP of 3.2 or less was 52.0% in the SSDM group and 44.3% in the control group, allowing for a 20% attrition rate.^46,47,48^ The minimum sample size created by the random number generator was 2204.

All end points were analyzed in a modified intent-to-treat (ITT) population that excluded patients with incorrect diagnoses (autoimmune diseases other than RA) upon enrollment. The missing values were imputed using multiple imputation by chained equations (mice) in R (eMethods in Supplement 2). Combined inferences from 5 imputed data sets were based on Rubin rules.^49^ All end points were also performed on per-protocol analysis. The primary end point was also analyzed using the worst-case scenario imputation, and the inverse probability of censoring weighted (IPCW) method.^50^ Continuous or discrete variables were defined as mean (SD) or median (IQR), and were compared between the 2 groups using the *t* test for normally distributed data and Wilcoxon rank-sum test for data that were not normally distributed. Categorical variables were analyzed using χ^2^ test and were shown as percentages. Preplanned subgroup analyses were conducted based on disease activity at baseline. All primary and secondary end points analyses were adjusted for center effect using the Cochran-Mantel-Haenszel or quantile regression. Other subgroup analyses were post hoc without adjustments. Statistical significance was set at 2-sided *P* < .05. All data analyses were conducted using the SAS version 9.4 (SAS Institute) and R version 4.2.1 (R package for statistical computing). Analysis was conducted from October 2020 to May 2022.

## Results

Of 3374 participants screened for eligibility, 2204 patients with RA were randomized, and 2197 patients (mean [SD] age, 50.5 [12.4] years; 1812 [82.5%] female) were followed up with, and there were 1099 patients in the SSDM group and 1098 patients in the control group. Demographic and clinical characteristics of the patients in the 2 groups were shown in Table 1. The dropout rate was 11.9% in the SSDM group vs 19.3% in the control group (difference between groups, 7.4%; 95% CI, 4.4% to 10.4%; *P* < .001). The mean (SD) adherence to the SSDM was 96.5% (10.2%).

**Table 1.** Baseline Characteristics of the Modified Intention-to-Treat Population

Characteristics	Median (IQR)	
	SSDM group (n = 1099)	Control group (n = 1098)
Sex, No. (%)		
Female	903 (82.2)	909 (82.8)
Male	196 (17.8)	189 (17.2)
Age, mean (SD), y	50.7 (12.4)	50.2 (12.5)
Disease duration, y	2.6 (1.7-8.2)	3.1 (1.8-8.5)
Educational background, No. (%)		
Secondary school or higher	650 (59.1)	627 (57.1)
Primary school	447 (40.7)	469 (42.7)
Unknown	2 (0.2)	2 (0.2)
DAS28-CRP, No. (%)	3.8 (1.4)	3.8 (1.4)
≤3.2	410 (37.3)	409 (37.2)
>3.2	689 (62.7)	689 (62.8)
No. of tender joints (0-28)	4 (1-8)	4 (1-8)
No. of swollen joints (0-28)	2 (0-4)	1 (0-4)
CDAI	14.4 (9.0-23.0)	14.0 (8.9-22.9)
SDAI	15.6 (9.5-25.1)	15.1 (9.7-25.1)
CRP, mg/L	3.1 (1.2-8.3)	3.3 (1.5-9.0)
PtGA score	47.0 (22.0-55.0)	48.0 (22.0-51.0)
PhGA score	42.5 (25.0-50.0)	45.0 (22.0-50.0)
mHAQ score	1 (0-5)	1 (0-5)
SF-36 PCS	45.1 (36.2-54.0)	40.0 (33.7-52.3)
SF-36 MCS	36.5 (30.5-41.8)	39.4 (30.7-43.2)
HADS		
Anxiety	6 (3-8)	6 (3-9)
Depression	6 (3-9)	6 (4-8)

Abbreviations: CDAI, Clinical Disease Activity Index; CRP, C-reactive protein; DAS28-CRP, 28-joint disease activity score using C-reactive protein; HADS, Hospital Anxiety and Depression Scale; MCS, mental component score; mHAQ, modified Health Assessment Questionnaire; PCS, physical component score; PhGA, physician global assessment of disease activity; PtGA, patient’s global assessment of disease activity; SDAI, Simplified Disease Activity Index; SF-36, the 36-Item Short Form Survey; SSDM, Smart System of Disease Management.

To convert CRP from mg/L to mg/dL, multiply by 10.

### Primary Outcome

At month 6, the rate of patients with a DAS28-CRP score of 3.2 or less, as determined by the modified ITT analysis after multiple imputation, was 71.0% (780 of 1099) in the SSDM group vs 64.5% (708 of 1098) in the control group (difference between groups, 6.6%; 95% CI, 2.7% to 10.4%; *P* = .001; Table 2). Statistically significant differences in the primary outcome were also evident in worst-case scenario imputation, IPCW analysis, and the per-protocol analysis (*P* = .05, eFigure 2 in Supplement 2).

**Table 2.** The Outcomes at Month 6 in the SSDM and Control Groups (ITT Analysis After Multiple Imputation and PP Analysis)

Outcomes	Population, median (IQR)							
	ITT				PP			
	SSDM group (n = 1099)	Control group (n = 1098)	Group difference (95% CI)^a^	*P* value	SSDM group (n = 968)	Control group (n = 886)	Group difference (95% CI)^a^	*P* value
DAS28-CRP	2.6 (2.0 to 3.3)	2.7 (2.0 to 3.5)	−0.1 (−0.2 to 0)	.007	2.6 (2.0 to 3.3)	2.7 (2.0 to 3.5)	−0.1 (−0.2 to 0)	.005
DAS28-CRP ≤ 3.2, No. (%)	780 (71.0)	708 (64.5)	6.6 (2.7 to 10.4)	.001	696 (71.9)	577 (65.1)	6.8 (2.7 to 10.9)	.001
Moderate-to-good EULAR response, No. (%)	640 (58.3)	580 (52.8)	5.6 (1.4 to 9.8)	.01	567 (58.6)	458 (51.7)	7.0 (2.5 to 11.4)	.002
CDAI	8.9 (5.0 to 12.2)	9.0 (5.0 to 12.7)	−0.1 (−0.8 to 0.6)	.75	8.5 (4.5 to 12.0)	8.8 (4.5 to 13.0)	0 (−0.3 to 0.3)	>.99
SDAI	9.6 (5.6 to 13.7)	9.8 (5.6 to 14.1)	− 0.3 (−1.1 to 0.5)	.45	9.2 (5.1 to 13.5)	9.5 (5.1 to 14.2)	−0.4 (−0.9 to 0.2)	.24
Tender joint counts	1 (0 to 3)	2 (0 to 4)	0	>.99	1 (0 to 2)	1 (0 to 4)	0	>.99
Swollen joint counts	0 (0 to 1)	0 (0 to1)	0	>.99	0 (0 to 1)	0 (0 to 1)	0	>.99
ACR/EULAR Boolean remission, No. (%)	115 (10.5)	87 (7.9)	2.0 (−0.3 to 4.3)	.09	112 (11.6)	81 (9.1)	2.4 (−0.3 to 5.0)	.08
RA flare, No. of total No (%)	51 of 410 (12.4)	61 of 409 (14.9)	−2.4 (−8.2 to 3.4)	.41	37 of 363 (10.2)	42 of 343 (12.2)	−2.2 (−6.8 to 2.4)	.35
PtGA	31.8 (16.8 to 50.0)	30.3 (17.4 to 50.0)	0	>.99	32.0 (16.0 to 50.0)	30.0 (17.0 to 50.0)	0 (−0.3 to 0.3)	>.99
PhGA	30.0 (10.2 to 50.0)	29.5 (14.0 to 50.0)	0	>.99	30.0 (16.0 to 50.0)	30.0 (16.0 to 50.0)	0 (−0.3 to 0.3)	>.99
SF-36 PCS	50.8 (42.7 to 56.6)	52.4 (43.6 to 58.0)	−1.6 (−3.3 to 0.2)	.07	51.2 (43.3 to 56.9)	53.0 (43.2 to 58.7)	−1.4 (−0.4 to −2.4)	.007
SF-36 MCS	37.3 (31.7 to 42.0)	38.3 (33.9 to 42.8)	−0.9 (−2.3 to 0.6)	.18	37.8 (32.6 to 42.2)	38.2 (34.4 to 42.9)	−0.4 (−1.3 to 0.4)	.32
HADS								
Anxiety	5 (2 to 8)	5 (1 to 7)	1 (0 to 2)	.004	5 (2 to 7)	4 (1 to 7)	0	>.99
Depression	6 (2 to 8)	5 (1 to 7)	1 (−1 to 2)	.38	5 (2 to 8)	4 (1 to 7)	0	>.99
mHAQ score	0 (0 to 2)	0 (0 to 2)	0	>.99	0 (0 to 2)	0 (0 to 2)	NA	NA

Abbreviations: ACR, American College of Rheumatology; CDAI, Clinical Disease Activity Index; DAS28-CRP, 28-joint disease activity score using C-reactive protein; EULAR, European League Against Rheumatism; HADS, Hospital Anxiety and Depression Scale; ITT, intention-to-treat; MCS, mental component score; mHAQ, modified Health Assessment Questionnaire; NA, not applicable; PCS, physical component score; PhGA, physician global assessment of disease activity; PP, per-protocol; PtGA, patient’s global assessment of disease activity; SDAI, Simplified Disease Activity Index; SF-36, the 36-Item Short Form Survey; SSDM, Smart System of Disease Management.

^a^ Differences are median differences or differences between proportions.

### Secondary Outcomes

The SSDM group had a higher moderate-to-good EULAR response rate (Table 2). In the 6-month extension phase, almost all end point measures improved significantly in both groups, including the rate of DAS28-CRP of 3.2 or less, moderate-to-good EULAR response rate, ACR/EULAR Boolean remission rate, and the change in CDAI and SDAI. The rate of patients with a DAS28-CRP of 3.2 or less in the control group increased from 65.1% at month 6 to 77.7% at month 12 (change from 6 months 12.7%; 95% CI, 8.6% to 16.8%; *P* < .001) in the per-protocol analysis (Table 3). Such a rate was comparable with that in the SSDM group (group difference −0.2%; 95% CI, −3.9% to 3.4%; *P* = .90). The median (IQR) numbers of outpatient visits were significantly higher in the SSDM group than in the control group (3 [2 to 6] vs 3 [2 to 4]; difference between groups, 1; 95% CI, 0 to 1; *P* < .001).

**Table 3.** The Extension Period From Month 6 to Month 12 in the Per-protocol Population

Outcome	SSDM group				Control group				Group difference (95% CI)^a^	*P* value^a^
	Month 6	Month 12	Change from month 6 (95% CI)^a^	*P* value	Month 6	Month 12	Change from month 6 (95% CI)	*P* value		
DAS28-CRP, median (IQR)	2.6 (2.0 to 3.3)	2.3 (1.7 to 3.1)	−0.2 (−0.3 to −0.1)	<.001	2.7 (2.0 to 3.5)	2.3 (1.7 to 3.1)	−0.3 (−0.4 to −0.2)	<.001	0.0 (−0.1 to 0.1)	>.99
DAS28-CRP ≤ 3.2, No. (%)	696 (71.9)	742 (78.2)	6.4 (2.5 to 10.2)	.001	577 (65.1)	668 (77.7)	12.7 (8.6 to 16.8)	<.001	−0.2 (−3.9 to 3.4)	.90
Moderate-to-good EULAR response, No. (%)	567 (58.6)	633 (66.7)	8.2 (3.9 to 12.4)	<.001	458 (51.7)	554 (64.4)	13.0 (8.5 to 17.5)	<.001	2.0 (−2.3 to 6.2)	.37
CDAI, median (IQR)	8.5 (4.5 to 12.0)	6.0 (3.4 to 10.0)	−1.5 (−1.9 to −1.1)	<.001	8.8 (4.5 to 13.0)	5.9 (3.4 to 10.0)	−1.5 (−1.8 to −1.2)	<.001	0 (−0.2 to 0.2)	>.99
SDAI, median (IQR)	9.2 (5.1 to 13.5)	6.8 (3.9 to 10.7)	−1.7 (−2.2 to −1.2)	<.001	9.5 (5.1 to 14.2)	6.6 (3.9 to 10.5)	−1.6 (−2.1 to −1.2)	<.001	0 (−0.3 to 0.3)	.95
Tender joint counts, median (IQR)	1 (0 to 2)	1 (0 to 2)	0	>.99	1 (0 to 4)	1 (0 to 2)	0	>.99	0	>.99
Swollen joint counts, median (IQR)	0 (0 to 1)	0 (0 to 1)	0	>.99	0 (0 to 1)	0 (0 to 1)	0	>.99	0	>.99
ACR/EULAR Boolean remission, No. (%)	112 (11.6)	140 (14.8)	3.2 (0.3 to 6.1)	<.001	81(9.1)	128 (14.9)	5.8 (2.9 to 8.7)	<.001	−0.9 (−3.8 to 2.1)	.57
PtGA score, median (IQR)	32.0 (16.0 to 50.0)	22.0 (12.0 to 37.0)	−3.0 (−3.7 to −2.3)	<.001	30.0 (17.0 to 50.0)	21.0 (12.0 to 37.0)	−2.0 (−2.6 to −1.4)	<.001	0 (−0.2 to 0.2)	>.99
PhGA score, median (IQR)	30.0 (16.0 to 50.0)	21.0 (11.0 to 37.0)	−3.0 (−3.7 to −2.3)	<.001	30.0 (16.0 to 50.0)	21.0 (10.0 to 37.0)	−3.0 (−3.6 to −2.4)	<.001	0	>.99
SF-36 PCS, median (IQR)	51.2 (43.3 to 56.9)	57.4 (51.2 to 61.3)	4.1 (2.9 to 5.3)	<.001	53.0 (43.2 to 58.7)	57.7 (52.9 to 60.9)	2.3 (1.3 to 3.2)	<.001	0.2 (−1.1 to 1.5)	.76
SF-36 MCS, median (IQR)	37.8 (32.6 to 42.2)	39.3 (34.9 to 42.5)	1.5 (0.8 to 2.2)	<.001	38.2 (34.4 to 42.9)	38.1 (34.9 to 41.2)	−0.9 (−0.1 to 1.9)	.07	−0.2 (−1.6 to 1.2)	.79
HADS, median (IQR)										
Anxiety	5 (2 to 7)	2 (0 to 6)	−1 (−2 to −1)	<.001	4 (1 to 7)	2 (0 to 6)	0	>.99	0	>.99
Depression	5 (2 to 8)	2 (0 to 6)	−1 (−1 to −1)	<.001	4 (1 to 7)	2 (0 to 7)	−1 (−1 to −1)	<.001	0	>.99
mHAQ score, median (IQR)	0 (0 to 2)	0 (0 to 1)	0	>.99	0 (0 to 2)	0	0	>.99	0	>.99

Abbreviations: ACR, American College of Rheumatology; CDAI, Clinical Disease Activity Index; DAS28-CRP, 28-joint disease activity score using C-reactive protein; EULAR, European League Against Rheumatism; HADS, Hospital Anxiety and Depression Scale; MCS, mental component score; mHAQ, modified Health Assessment Questionnaire; PCS, physical component score; PhGA, Physician global assessment of disease activity; PP, per-protocol; PtGA, Patient’s global assessment of disease activity; SDAI, Simplified Disease Activity Index; SF-36, the 36-Item Short Form Survey; SSDM, Smart System of Disease Management.

^a^ Comparisons were done between the SSDM group and control group at month 12. Differences are median differences or differences between proportions.

### Subgroup Analysis and Rheumatologist Intervention

**Figure 2.**
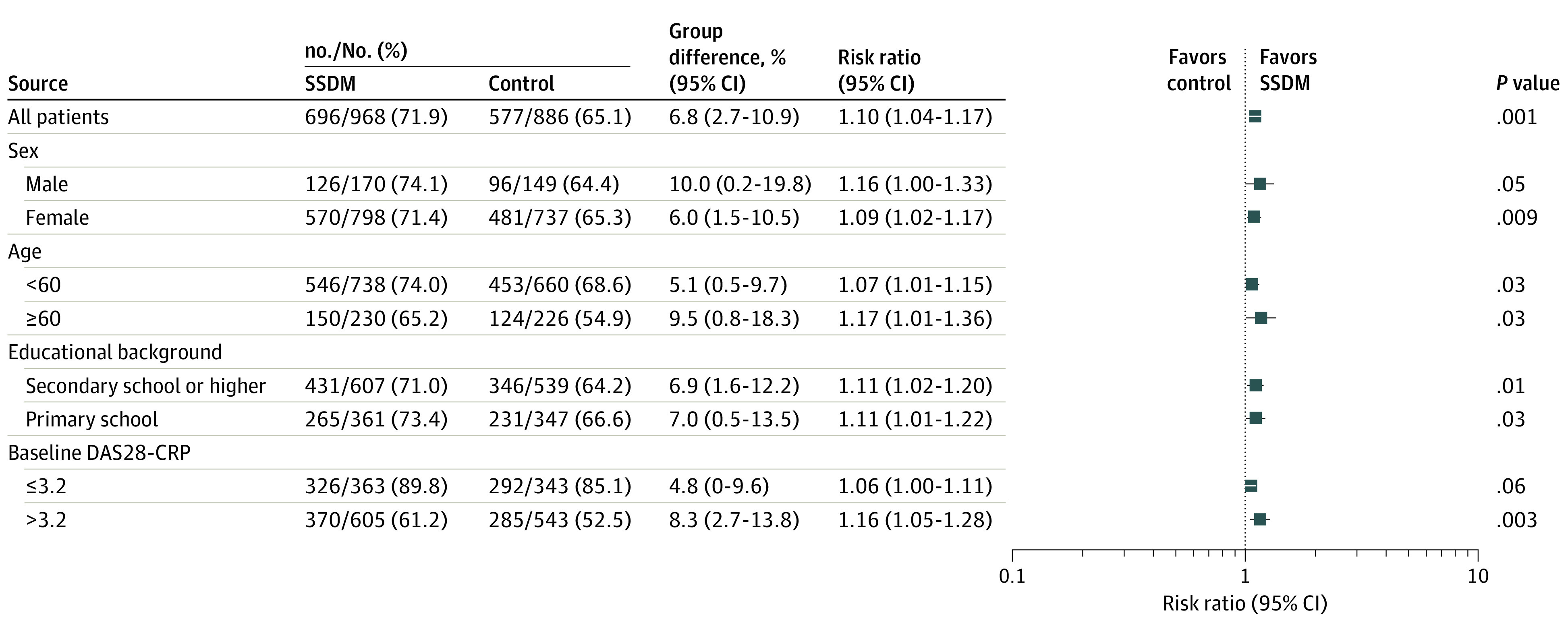
Subgroup Analysis in Per-protocol Analysis DAS28-CRP indicates disease activity score in 28 joints in C-reactive protein; SSDM, Smart System of Disease Management.

In the subgroup analysis, the rate of patients with a DAS28-CRP of 3.2 or less at month 6 was higher in the SSDM group regardless of age, sex, and education in the per-protocol analysis (Figure 2). Further analysis that separated baseline disease activity into 4 statuses (REM, LDA, MDA, and HDA) suggested distinct patterns in disease progression. In patients with MDA at baseline, the percentage of patients with a DAS28-CRP of 3.2 or less at month 6 was higher in the SSDM group than in the control group (difference between groups, 8.1%; 95% CI, 1.5% to 14.6%; *P* = .02) (eTable 2 in Supplement 2). In patients with LDA at baseline, the rate of deterioration (DAS28-CRP > 3.2) at month 6 was lower in SSDM group than in the control group (difference between groups, −13.4%; 95% CI, −22.5% to −4.3%; *P* = .004) (eTable 3 in Supplement 2).

A total of 226 alerts were noted in 202 patients in the SSDM group in the initial phase. Among patients with alerts, the rate of DAS28-CRP of 3.2 or less at month 6 was 76.9% in patients with rheumatologist intervention and 63.7% in patients without intervention (difference between groups, 13.2%; 95% CI, 0.6% to 25.8%; *P* = .048). A total of 1247 alerts were noted in 989 patients in the extension period. The rate of DAS28-CRP of 3.2 or less at month 12 was 82.9% in patients with rheumatologist intervention and 55.9% in patients without intervention (difference between groups, 27.0%; 95% CI, 20.3% to 33.3%; *P* < .001; eTable 4 in Supplement 2). The overall response rate of investigators to alerts was 22.8%. Patients with intervention showed more changes in medication at month 12 (eTable 5 in Supplement 2). The rates of DAS28-CRP of 3.2 or less in patients with multiple alerts were shown in eTable 6 in Supplement 2.

### Adverse Events

None of the adverse events were related to the intervention of digital health application. The reported adverse events reported were shown in eTable 7 in Supplement 2.

## Discussion

This randomized clinical trial demonstrated a higher rate of patients with a DAS28-CRP score of 3.2 or less at month 6 in the SSDM group than in the conventional care control group. The observed difference between the 2 groups was supported by the results of sensitivity analyses using the IPCW and per-protocol analysis. Switching to SSDM in the patients randomized into the control group in the initial phase resulted in a comparable rate of patients with a DAS28-CRP of 3.2 or less at the end of the extension period. The rate of patients with a DAS28-CRP of 3.2 or less was also higher in the SSDM group in all subgroup analyses based on age and educational level, suggesting older age and low educational level (as long as the patients were literate) are not significant barriers to using SSDM to manage their disease.

The impact of digital applications on electronic PROs (ePROs) has been examined in several previous studies.^28,30,51^ The applications could facilitate routine PRO collection and the use of ePROs in clinical care for RA.^52^ The adherence to the ePRO application, if properly designed, was also high.^29,51^ In addition to electronic data collection, patient-rheumatologist interaction contributed to shared decision-making and physician awareness of disease fluctuations.^53^ The ability of the web-based application intervention feature to report symptom status in our study also resulted in clinical improvement in disease activity in patients with RA.

There are at least 2 factors that may contribute to the effectiveness of the smartphone application in RA disease control. First, a higher number of outpatient visits were observed in the SSDM group. It is likely that using SSDM per se increases patients’ awareness of health, which in turn brings them back to rheumatologists more often. More frequent visits contributing to better disease control was also supported by other chronic diseases (eg, hypertension).^54,55^ Second, the application-based alert and intervention allow physicians to be aware of the need for prompt intervention and motivate patients to manage their disease.

To our knowledge, this study was the largest randomized clinical trial to identify the validity of application-based RA management. Smartphone applications, such as SSDM, could be used in daily clinical practice to reduce the management burden of rheumatologists. The inclusion of patients with a DAS28-CRP of 3.2 or less at baseline could increase the generalizability of this study. Although the inclusion may diminish the effect size of this study, these patients represent a large subset of patients in a daily practice setting (approximately 40% of the study population in this trial). As such, the inclusion of these patients is important from a clinical perspective, particularly for measures (such as SSDM in this trial) that are more likely to be used in patients with low disease activity or at remission. The results of our study suggest that the SSDM system has the potential to serve as a supplementary platform for reporting adverse events, confirming the findings of previous research.^56,57^ The findings in this trial are also important in an era of novel public health threats exemplified by the ongoing COVID-19 pandemic and its impact on the behaviors of patients and physicians.^58^ Virtual visits or telemedicine need to be proven as effective as outpatient clinic visits in controlling the disease activity of RA.^59^

### Limitations

This study has limitations. First, the attrition rate differed between the 2 groups, which may bias the results. The sensitivity analysis using the worst-case scenario, per-protocol analysis, and IPCW analysis were introduced to the modified ITT analysis to overcome the attrition bias. Second, laboratory testing must be conducted to obtain the DAS28-CRP score. Whether patient self-assessment that does not require laboratory testing (eg, CDAI and Routine Assessment of Patient Index Data 3) could be developed into digital applications for clinical use is unknown. Third, cluster randomization is a more appropriate design due to the minimization of communication between the patients as well as modification of physician behavior, which make it harder to get significant effects. However, individual randomization could reduce treatment bias between study centers.

## Conclusions

In this randomized clinical trial of patients with RA, the use of digital health applications to assess patient-reported outcomes increased the rate of patients with a DAS28-CRP score of 3.2 or less at month 6. This study provides modest clinical value that application-based patient-reported outcomes and intervention could be an effective way to treat patients with RA and may provide evidence for diseases with complex treatment targets.

## Notes

Supplement 1.
Trial Protocol

Supplement 2. **eTable 1.** Study Inclusion and Exclusion Criteria
eMethods.
**eTable 2. **Subgroup Analysis of Patients With DAS28-CRP ≤ 3.2 at Month 6 in the Per-Protocol Analysis **eTable 3. **Subgroup Analysis of Patients With DAS28-CRP > 3.2 at Month 6 in the Per-Protocol Analysis **eTable 4. **Comparison of Outcomes at Month 6 and Month 12 in Patients With or Without Intervention Upon Alert From SSDM **eTable 5. **The Change in Treatment Between Patients With or Without Intervention **eTable 6. **The Rate of DAS28-CRP ≤ 3.2 Among Participants With Alerts and Intervention **eTable 7.** Safety Data From Baseline to Month 6 **eFigure.** The Rate of Patients With DAS28-CRP ≤ 3.2 at Month 6

Supplement 3.
Data Sharing Statement
