# Error in Results and Figure 1

**DOI:** 10.1001/jamanetworkopen.2023.16007

**Published:** 2023-05-19

In the Original Investigation titled “Management of Rheumatoid Arthritis With a Digital Health Application: A Multicenter, Pragmatic Randomized Clinical Trial,”^1^ published April 14, 2023, there was an error in the *P* value presented in the final sentence of the Primary Outcome subsection of the Results section and an error in the number of people allocated to the Smart System of Disease Management (SSDM) group who did not complete the trial in Figure 1. The *P* value should have read *P* ≤ .001, and the number of people excluded in the SSDM group should have read 150. This article has been corrected.^1^
