# Supplementary material for: Management of Rheumatoid Arthritis With a Digital Health Application: A Multicenter, Pragmatic Randomized Clinical Trial
Source: JAMA Netw Open. 2023 Apr 14;6(4):e238343. doi: 10.1001/jamanetworkopen.2023.8343 (PMC10105314; doi:10.1001/jamanetworkopen.2023.8343)
Supplement: Supplement 1. — Trial Protocol [file jamanetwopen-e238343-s001.pdf]

Management of rheumatoid arthritis with a mobile health application: A multi-center, pragmatic randomized controlled trial

This supplement contains the following items

Study Protocol 2
Protocol Amendment 11
Statistical Analysis Plan 12
Appendix 1. Evaluation of Disease Activity 16
  1. DAS28-CRP 16
  2. EULAR response criteria 16
  3. ACR/EULAR Boolean remission 16
  4. Clinical Disease Activity Index (CDAI) 16
  5. Simple Disease Activity Index (SDAI) 17
  6. Patient's global assessment of disease activity (PtGA) 17
  7. Physician's global assessment of disease activity (PhGA) 17
Appendix 2. 2010 ACR/EULAR RA classification criteria 19
Appendix 3. 36-Item Short Form Survey Instrument (SF-36) 19
Appendix 4. The modified Health Assessment Questionnaire Disability index 22

Study Protocol

A Randomized Controlled Multicenter Study of Rheumatoid Arthritis Disease Activity Assessment Based on Smart System of Disease Management (SSDM)

Principal Investigator: Li Zhanguo
Research Institution: Peking University People's Hospital
Department: Rheumatology and Immunology
Contact: Li Zhanguo, mobile phone: +8613910713924
Li Chun, mobile phone: +8613811190098

1. Signature Page

For a multi-center study, the signatures of the investigators in the designing of the study protocol indicate that the protocol has been read and approved, and the relevant contents of the protocol have been confirmed.

The information of the clinical investigators on the signature page should include the name, position, title and department.

Center	Department	Name	Academic title	Job Title	Signature
Peking University People's Hospital	Rheumatology	Li Zhanguo	Professor	Director	
Peking University People's Hospital	Rheumatology	Li Chun	Associate professor	/	
Anhui Provincial Hospital	Rheumatology	Li Xiaomei	Professor	Director	
The First Affiliated Hospital of Anhui Medical University	Rheumatology	Shuai Zongwen	Professor	Director	
Peking University International Hospital	Rheumatology	Li Shengguang	Professor	Director	
Xuanwu Hospital of Beijing Capital Medical University	Rheumatology	Zhao Yi	Professor	Director	
Dongguan Donghua Hospital, Guangdong Province	Rheumatology	Wu Henglian	Professor	Director	
The Sixth Affiliated Hospital of Guangdong Sun Yat-sen University	Rheumatology	Huang Jianlin	Professor	Director	
Hebei Provincial People's Hospital	Rheumatology	Zhang Fengxiao	Professor	Director	
Bethune International Peace Hospital of Hebei Province	Rheumatology	Li Zhenbin	Professor	Director	
The Second Xiangya Hospital of Central South University	Rheumatology	Li Fen	Professor	Director	
Jiangsu Provincial People's Hospital	Rheumatology	Zhang Miaojia	Professor	Director	
Subei People's Hospital of Jiangsu Province	Rheumatology	Wei Hua	Professor	Director	
The First Affiliated Hospital of Baotou Medical College, Inner Mongolia	Rheumatology	Wang Yongfu	Professor	Director	
Linyi People's Hospital of Shandong Province	Rheumatology	Zhang Zhenchun	Professor	Director	
Shandong Provincial Hospital	Rheumatology	Sun Hongsheng	Professor	Director	
Mianyang Central Hospital, Sichuan Province	Rheumatology	Yang Jing	Professor	Director	
Tianjin Medical University General Hospital	Rheumatology	Wei Wei	Professor	Director	
Yunnan Provincial People's Hospital	Rheumatology	Li Qin	Professor	Director	
Second Affiliated Hospital of Zhejiang University School of Medicine	Rheumatology	Wu Huaxiang	Professor	Director	
Zhejiang Provincial People's Hospital	Rheumatology	Li Yasong	Professor	Director	
Xijing Hospital	Rheumatology	Wu Zhenbiao	Professor	Director	
Tianjin First Central Hospital	Rheumatology	Qi Wufang	Professor	Director	

2. Abstract

2.1 Background

The Smart System of Disease Management (SSDM) is a series of application software developed by rheumatologists, rheumatoid arthritis (RA) patients, and Shanghai Gothic Internet Technology (Shanghai, China) for patients with chronic diseases. Preliminary studies have shown that the application of SSDM can increase the rate of RA patients with low disease activity (LDA, DAS28 ≤ 3.2), decrease the incidence of adverse events and reduce health care costs with high satisfaction. Therefore, the rate of clinical LDA in RA patients can be improved after regularly self-assessment using SSDM.

2.2 Purpose

To identify the rate of patients in LDA by using SSDM at month 6.

2.3 Method

This is a multicenter, pragmatic randomized controlled trial. The investigators at each center will screen potentially eligible participants, explain the trial to them, check inclusion and exclusion criteria, and obtain informed consent. RA patients who sign the informed consent were evaluated for disease activity using the 28-joint disease activity score (DAS28-CRP) at baseline. According to DAS28-CRP, participants will be divided into three groups (1) Remission (REM, DAS28-CRP ≤ 2.6) / Low Disease Activity (LDA, DAS28-CRP 2.6 - 3.2) group; (2) Moderate Disease Activity (MDA, DAS28-CRP 3.2 - 5.1) group; (3) High Disease Activity (HDA, DAS28-CRP > 5.1) group. Eligible patients will be randomly assigned at a 1:1 ratio into a SSDM group vs a conventional care control group.

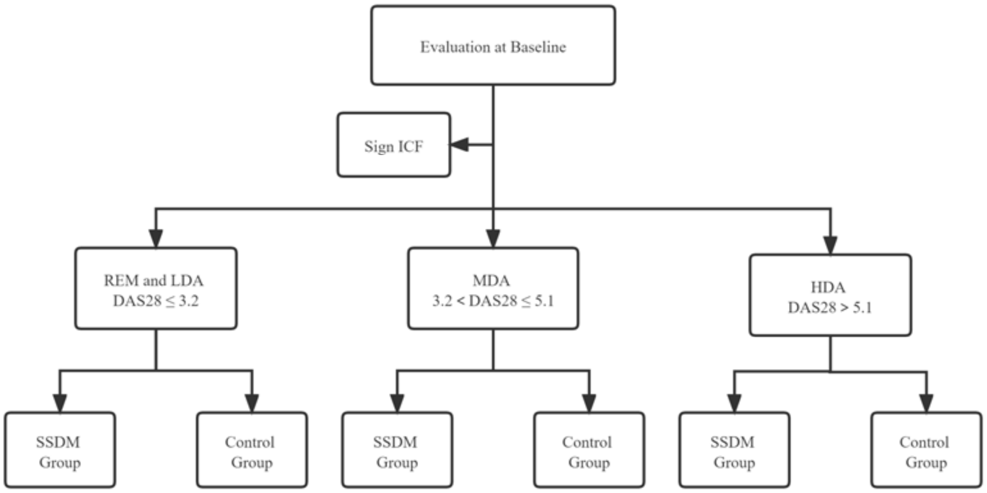

Figure 1. The randomization of this study

Patients in the SSDM group will watch a 15-minute video that describe the key features of the SSDM to allow them to correctly use of the app. Patients will conduct self-assessment and report the results once every month for 6 months. A 6-month extension period during which subjects in the conventional care group in the initial phase will be invited to switch to SSDM group, and patients in the SSDM group will continue to use SSDM for self-management.

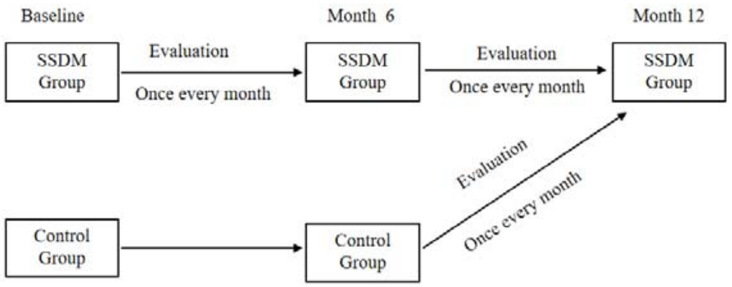

Figure 2. The design of this study

3. Study protocol

3.1 Background

Rheumatoid arthritis (RA) is a systemic autoimmune disease characterized by persistent synovitis, systemic inflammation and autoantibodies. The prevalence of RA in China is 0.28% and there are more than 5 million patients in China. In the absence of treatment, RA can lead to functional and work disability, premature mortality. In RA patients, bone erosion and cartilage destruction may be seen within the first 2 years of the disease. DAS28-CRP $\leq$ 3.2 are the treatment target of RA.

In China, there is a significant deficit of rheumatologists. It is impossible to assess disease activity for every patient in routine practices. Meanwhile, there are no follow-up activities after these patients leaving the clinic. The window of opportunity for closely control of disease activities was easily missed.

The Smart System of Disease Management (SSDM) is a series of application software developed for patients with chronic diseases. The SSDM has iOS, Android, and Web interfaces. The functions of patient platform include evaluation, treatment, lab report, evaluation report, knowledge, and chart. The disease assessment system adopts visualization technology. Patient can complete the tender and swollen joints evaluation on the touch screen. The system automatically generates a DAS28-CRP score, and demonstrates the conditions of patients in the form of curves and charts. The self-assessment of patients can be synchronized with the physician's platform in real time. Doctors can view the data of their patients through smart phones in real time, and perform statistical analysis on the patients' data.

From 2014 to 2015, we conducted a study on the feasibility and influential factors in performing self-assessment of DAS28-CRP with SSDM in Chinese RA patients. The study confirmed that RA patients were able to perform accurate self-assessment on DAS28-CRP. The reliability of the self-assessment using SSDM reached 86%.

SSDM has been applied in rheumatology for 4 years. The preliminary cohort study results showed that the application of SSDM could improve the rate of RA patients with REM/LDA, decrease the incidence of adverse events and reduce health care costs with high satisfaction. Self-management is recommended for patients with inflammatory arthritis, patients took an active role in their disease management. In an 8-year self-management project for patients with arthritis, self-management led to long-term maintenance of self-efficacy, psychological well-being and self-management techniques.[1] In patients with RA or psoriatic arthritis (PsA) treated with MTX, the patient's self-management could lead to significant reductions in healthcare, while maintaining clinical and psychosocial well-being.[2] Self-assessment of disease activity in RA patients led by nurses showed more frequently changes in DMARD therapy.[3] Smartphone as a tool for self-assessment and rehabilitation have been widely used in the rehabilitation of heart failure,[4] asthma,[5] diabetes[6] and lung diseases.[7] Therefore, smartphones are likely to become a tool for self-management in RA patients. In a survey of RA patients, 94% of RA patients believed that smartphone could have a more active role in self-management and reported it would be useful to develop a RA self-management application.[8] Therefore, we conducted a national multi-center, randomized controlled trial to evaluate the clinical significance of self-assessment with SSDM in RA patients.

3.2 Purpose

The purpose of this study was to evaluate the rate of patients with DAS28-CRP ≤ 3.2 by using SSDM at month 6.

3.3 Study Design

(1) Subjects, inclusion criteria, exclusion criteria, elimination criteria, withdrawal criteria, etc.

Subjects: All patients diagnosed with RA who were treated at Peking University People's Hospital and other clinical trial centers.

Inclusion criteria

1) Diagnosis of RA by investigators according to the 2010 American College of Rheumatology (ACR)/European League Against Rheumatism (EULAR) criteria

2) ≥ 18 years old

3) Have a smartphone

4) Able to evaluate disease activity by themselves

Exclusion criteria

1) Not able to read and use smartphone

2) Not able to perform the joint examination by themselves

3) Patients with cognitive impairment or mental condition which make them unable to comply with the requirements of self-management

4) Hand deformations or any other conditions (e.g., Parkinson's disease and late-stage Alzheimer's disease) that affect data input

5) Do not want to accept the treatment of RA or do not plan on receiving follow-up care at the research centers

6) Already a user of SSDM

Elimination criteria: Patients with incomplete clinical data.

Withdrawal criteria: Patients who request to withdraw from the clinical trial.

(2) Randomization of subjects, whether to set up a control group

1) Randomization

Following the evaluation by rheumatologists, eligible participants will be randomized at a 1:1 ratio to a SSDM group vs a conventional care control group. The randomization sequence was generated with an interactive web response system (BioVoicer Technology Co. Ltd, Beijing, China) using a block design (block sizes of 4). The randomization was stratified based on the DAS28-CRP score at the baseline (≤ 3.2, 3.2 - 5.1, or > 5.1). Concealment was conducted centrally using an interactive web response system.

2) Intervention

① SSDM intervention group: Upon the first use of the system, the following information must be entered with the help of a research staff: full name, sex, date of birth, date of initial diagnosis, comorbidities, education level, occupation, family income, annual medical expenses and DAS28-CRP at each research site. Other information that entered included: 1) lab results (e.g., routine blood test, liver and kidney function, erythrocyte sedimentation rate, CRP), submitted as photographs and automatically processed to extract key information via Optical Character Recognition technology; 2) medications, for RA as well as co-morbid conditions; and 3) perceived adverse reactions (a total of 33 types of symptoms).

Patients randomized to the SSDM group watch a 15-minute video that described the key features of SSDM to allow correct use of the app. After learning, the patients will do the self-assessment all by themselves using SSDM. If there is inconsistency between patient and physician, the physician will correct it and then the patient will do it again. At last, the use of SSDM by patients and self-assessment

of DAS28-CRP were confirmed by physicians.

② Conventional care control group: Patients randomized to the control group will receive conventional care, and should come back for office visits at month 6 and month 12. The information of baseline characteristics will also be collected.

All the primary and secondary endpoint will be assessed by rheumatologists.

(3) Intervention measures

Duration of the Study: 12 Months

(4) Follow-up Plan

Period	Screening	Follow-up Period											
Time (Month)	0	1	2	3	4	5	6	7	8	9	10	11	12
Follow-up(day)	0	30	60	90	120	150	180	210	240	270	300	330	360
ICF signing	×												
Inclusion/exclusion criteria	×												
Demographic data	×												
DAS28-CRP	×	×	×	×	×	×	×	×	×	×	×	×	×
CDAI	×	×	×	×	×	×	×	×	×	×	×	×	×
SDAI	×	×	×	×	×	×	×	×	×	×	×	×	×
HAQ	×	×	×	×	×	×	×	×	×	×	×	×	×
SF-36	×	×	×	×	×	×	×	×	×	×	×	×	×
HADS	×	×	×	×	×	×	×	×	×	×	×	×	×
Morning stiffness	×	×	×	×	×	×	×	×	×	×	×	×	×
Medication	×	×	×	×	×	×	×	×	×	×	×	×	×

(5) Selection and confirmation of primary measurement indicators or outcome indicators, secondary observation endpoints

1) The Primary Endpoint

The primary endpoint is the rate of DAS28-CRP ≤ 3.2 at month 6, as assessed by rheumatologists

2) The Secondary Endpoint

① The rate of DAS28-CRP ≤ 3.2 between the two groups at month 12, assessed by rheumatologists.

② The RA flare rate between the two groups at month 6 and 12.

③ The proportion of patients with moderate-to-good EULAR response between the two groups at month 6 and12.

④ ACR/EULAR Boolean remission rate between the two groups at month 6 and 12.

⑤ The HADS between the two groups at month 6 and 12.

⑥ The SF-36 between the two groups at month 6 and 12.

⑦ Clinical disease activity index (CDAI) between the two groups at month 6 and 12.

⑧ The numbers of tender joint counts and swollen joint counts between the two groups at month 6 and 12.

⑨ The effect of active intervention by doctors between the two groups at month 6 and 12.

⑩ The number and rate of adverse events, either reported by the rheumatologists or resulting from an alert in the SSDM.

(6) Sample Size

Sample size calculation 1) 6-month disease control rate of 44.3% in the control group and 52% in the SSDM group; 2) 2-sided α at 0·05 and power at 90%; 3) 20% attrition rate. The calculation yielded a total of 2200 patients. And 2204 were the minimum sample size created by the Random Number Generator.

(7) The enrollment period, whether to design an interim analysis

The enrollment is expected to be half a year.

3.4 Previous results

(1) We verified the feasibility and influential factors in performing self-evaluation on DAS28-CRP with SSDM by RA patients from December 14, 2014 to February 2, 2015. This study included two phases. In the first phase, 55 cases were enrolled. The intra-class correlation coefficient (ICC) of tender joint counts (TJCs) and DAS28-CRP scores were 0.93 and 0.94, but the ICC of swollen joint counts (SJCs) was 0.68. The analysis showed that the duration of disease was the only associate factor with significant difference ($P < .05$). In the second phase, the education courses were more emphasized in identification of swollen joints. As a result, the ICCs of SJCs, TJCs and DAS28-CRP were improved to 0.93, 0.93 and 0.92, respectively. RA patients could master SSDM and perform accurate self-evaluation on DAS28-CRP and HAQ.

(2) Another study evaluated the patterns of T2T and related influential factors among RA patients after applying SSDM in real world. From June 2014 to June 2017, 1,232 RA patients from 145 hospitals across China were followed up for more than 6 months through SSDM. The rate of patients in REM/LDA was 40% (491/1,232) at baseline, and improved significantly to 64% (793/1,232) after 6 month follow up. Among patients in REM/LDA at baseline, 75% (369/491) maintained LDA, 25% (122/491) relapsed. Compared with relapsers, patients with LDA performed more self-evaluation (5.78 vs 4.73, $P = .017$), higher rate on DMARDs (69% vs 59%), lower rate on NSAIDs (4% vs 13%, $P < .01$) or glucocorticoid (3% vs 8%, $P < .01$). Among patients failed to reach REM/LDA at baseline, 57% (424/741) achieved REM/LDA after 6 months. Comparing with 6 month failure (317/741), new REM/LDA achiever got shorter morning stiffness time (16.77 ± 27.12 vs 27.03 ± 33.76 minutes, $P < .001$), lower HAQ score (2.17 ± 3.36 vs 3.36 ± 4.05, $P < .001$) at baseline, performed more self-evaluation (6.56 vs 5.35, $P = .007$). However, even in patients of 6 month failure, the morning stiffness time and HAQ score improved significantly in final follow up comparing with those at baseline (16.59 ± 28.76 vs 27.03 ± 33.76, $P < .001$; 2.77 ± 3.52 vs 3.36 ± 4.05, $P < .001$) .

3.5 Clinical Study Site and Data Management

The study does not involve sample collection, storage sites and laboratory research sites.

The data in this study are divided into electronic data and paper data. The paper data should be archived and stored in the order of labeling for inspection. Electronic data files include databases, inspection procedures, analysis procedures, analysis results, codebooks and instruction documents, etc., which should be stored in categories. Multiple backups of electronic data should be stored on different disks or recording media to prevent damage. All original data should be kept within the required time limit.

Electronic data is stored on the Alibaba Cloud. The security of the data is protected by the corresponding laws and regulations.

3.6 Statistical analysis

All endpoints will be analyzed in a modified intent-to-treat (ITT) population that excluded patients with incorrect diagnosis (autoimmune diseases other than RA) upon enrolment. Sensitivity analysis will be performed to assess the robustness of the primary analysis using multiple imputation, last observation carried forward imputation, and the worst case scenario imputation. The primary endpoint will also be analysed using the per-protocol (PP) analysis, inverse probability of censoring weighted (IPCW) method. Continuous variables are defined as mean (standard deviation, SD) or median (inter-quartile range, IQR) and will be compared between the two groups using the Student's t-test or Wilcoxon

rank-sum test. Categorical variables are defined as frequency or percentage and compared between the two groups using $\chi 2$ or Wilcoxon rank sum test, as appropriate. The trend of changes in DAS28-CRP and EULAR responses was calculated from baseline to month 6. Preplanned subgroup analyses will be conducted based on disease activity at baseline. Other subgroup analyses were post hoc. Statistical significance are set at 2-sided $P < .05$. All data analyses are conducted using the using the SAS and R.

3.7 Safety Evaluation

This study does not involve safety issue, so safety evaluation is not required. This study will collect adverse events on the treatment of RA.

3.8 Subject Protection

This clinical trial should not start until the clinical trial protocol and the informed consent form of the subjects have been submitted and approved by the Ethics Committee of Peking University People's Hospital. If the protocol is revised, only the corresponding revised part and the revised informed consent can be implemented after being reviewed and approved by the ethics committee, and a copy of the approval by the ethics committee of Peking University People's Hospital is required to be provided to the clinical inspector. If the protocol is revised to reduce the obvious risk of the subjects, it can be implemented immediately, but it must be submitted to the relevant departments and the ethics committee for the record as soon as possible.

Subjects can obtain information on RA disease knowledge and free online learning of RA knowledge, and get a free consultation.

The investigators keep the subject's evaluation results confidential. In the study, the identity information of the subjects was de-identified. The patients in the conventional treatment group used initials instead of the real names of the patients. The SSDM group can use the users' name (non-real name) for registration and subsequent login. All data is only used in this study.

3.9 Management of Clinical Study

If the protocol is revised, only the corresponding revised part and the revised informed consent can be implemented after being reviewed and approved by the ethics committee, and a copy of the approval by the ethics committee of Peking University People's Hospital is required to be provided to the clinical inspector. If the protocol is revised to reduce the obvious risk of the subjects, it can be implemented immediately, but it must be submitted to the relevant departments and the ethics committee for the record as soon as possible.

If severe safety issues arise during the trial, or major mistakes in the clinical trial protocol were found during the trial, the sponsor or the administrative department should request termination or the trial.

3.10 Clinical Trial Centers

(1) Peking University People's Hospital;
(2) Anhui Provincial Hospital;
(3) The First Affiliated Hospital of Anhui Medical University;
(4) Peking University International Hospital;
(5) Xuanwu Hospital of Beijing Capital Medical University;
(6) Donghua Hospital of Dongguan, Guangdong;
(7) The Sixth Affiliated Hospital of Guangdong Sun Yat-sen University;
(8) Hebei Provincial People's Hospital;
(9) Hebei Bethune International Peace Hospital;
(10) The Second Xiangya Hospital of Central South University;
(11) Jiangsu Provincial People's Hospital;
(12) Subei People's Hospital of Jiangsu Province;
(13) The First Affiliated Hospital of Baotou Medical College, Inner Mongolia;
(14) Linyi City People's Hospital of Shandong Province;
(15) Shandong Provincial Hospital;
(16) Mianyang Central Hospital of Sichuan Province;
(17) General Hospital of Tianjin Medical University;
(18) Yunnan Provincial People's Hospital;
(19) The Second Affiliated Hospital of Zhejiang University School of Medicine;
(20) Zhejiang Provincial People's Hospital.
(21) Xijing Hospital

(22) Tianjin First Central Hospital

References

1. Barlow J, Turner A, Swaby L, Gilchrist M, Wright C, Doherty M. An 8-yr follow-up of arthritis self-management programme participants.*Rheumatology (Oxford)*. 2009;48(2):128-33. doi:10.1093/rheumatology/ken429

2. Mcbain H, Shipley M, Olaleye A, Moore S, Newman S. A patient-initiated DMARD self-monitoring service for people with rheumatoid or psoriatic arthritis on methotrexate: a randomised controlled trial. *Ann Rheum Dis*. 2016;75(7):1343-9. doi:10.1136/annrheumdis-2015-207768

3. Dougados M, Soubrier M, Perrodeau E, et al. Impact of a nurse-led programme on comorbidity management and impact of a patient self-assessment of disease activity on the management of rheumatoid arthritis: results of a prospective, multicentre, randomised, controlled trial (COMEDRA). *Ann Rheum Dis*. 2015;74(9):1725-33. doi:10.1136/annrheumdis-2013-204733

4. Dang S, Karanam C, Gomez-Marin O. Outcomes of a Mobile Phone Intervention for Heart Failure in a Minority County Hospital Population. *Telemed J E Health*. 2017;23(6):473-484. doi:10.1089/tmj.2016.0211

5. Chan YY, Wang P, Rogers L, et al. The Asthma Mobile Health Study, a large-scale clinical observational study using ResearchKit. *Nat Biotechnol*. 2017;35(4):354-362. doi:10.1038/nbt.3826

6. Krishna S, Boren SA, Balas EA. Healthcare via cell phones: a systematic review. *Telemed J E Health*. 2009;15(3):231-40. doi:10.1089/tmj.2008.0099

7. Marshall A, Medvedev O, Antonov A. Use of a smartphone for improved self-management of pulmonary rehabilitation. *Int J Telemed Appl*. 2008; 2008:753064. doi:10.1155/2008/753064

8. Azevedo R, Bernardes M, Fonseca J, Lima A. Smartphone application for rheumatoid arthritis self-management: cross-sectional study revealed the usefulness, willingness to use and patients' needs. *Rheumatol Int*. 2015;35(10):1675-85. doi:10.1007/s00296-015-3270-9

Protocol Amendment

Amendment 1 19.04.12 The alert function has been added to the protocol.

In Brief, A red flag was raised upon one or more of the following conditions:

1. Disease activity exacerbation: the DAS28-CRP score increased to and remained at 3.2-5.1 (moderate disease activity, MDA) for 3 months, or increased to > 5.1 (high disease activity, HDA) in patients with remission (REM, DAS28-CRP ≤ 2.6) or low disease activity (LDA, > 2.6 but ≤ 3.2) at baseline;
2. Sustained MDA or worsening HDA: the DAS28-CRP score remained at 3.2-5.1 for 3 months, or increased to > 5.1 in patients with MDA at baseline;
3. HDA status: the DAS28-CRP score remained at > 5.1 for 3 months, decreased to 3.2-5.1 but with subsequent exacerbation (DAS28-CRP increase to > 5.1) at any time point or no further reduction by at least 1.2 within 3 months in patients with HDA at baseline.
4. The alert was also triggered upon elevated alanine aminotransferase or aspartate aminotransferase levels above 2-times the upper normal limit or a white blood cell count of < 2,000 or > 10,000 per mL.

Management of rheumatoid arthritis with a mobile health application: A multi-center, pragmatic randomized controlled trial

Statistical Analysis Plan

Analysis Summary

1. Study Title

Management of rheumatoid arthritis (RA) with a mobile health application: A multi-center, pragmatic randomized controlled trial

2. Primary Objectives

The primary outcome was the rate of patients with DAS28-CRP ≤ 3.2 at month 6

3. Secondary Objectives

(1) The proportion of patients with moderate-to-good EULAR response rate at month 6

(2) ACR/EULAR Boolean remission rate at month 6 and month 12

(3) The change in simplified disease activity index (SDAI) at month 6 and month 12

(4) The change in clinical disease activity index (CDAI) at month 6 and month 12

(5) The change in tender joint counts at month 6 and month 12

(6) The change in swollen joint counts at month 6 and month 12

(7) The change in Hospital Anxiety and Depression Scale (HADS) at month 6 and month 12

(8) The change in the 36-Item Short Form Survey (SF-36) at month 6 and month 12

(9) Flare rate at month 6 and month 12 at month 6 and month 12

(10) The rate of patients achieving DAS28-CRP-CRP ≤ 3.2 at month 12

(11) The numbers and rates of adverse events, either reported by the rheumatologists or resulting from an alert in the SSDM.

4. Patient Population

All patients diagnosed with RA who are treated at Peking University People's Hospital and other clinical trial centers.

4.1 Inclusion criteria:

(1) Diagnosis of RA by investigators according to the 2010 American College of Rheumatology (ACR)/European League Against Rheumatism (EULAR) criteria

(2) ≥ 18 years old

(3) Have a smartphone

(4) Able to evaluate disease activity by themselves

4.2 Exclusion criteria

(1) Not able to read and use smartphone

(2) Not able to perform the joint examination by themselves

(3) Patients with cognitive impairment or mental condition which make them unable to comply with the requirements of self-management

(4) Hand deformations or any other conditions (e.g., Parkinson's disease and late-stage Alzheimer's disease) that affect data input

(5) Do not want to accept the treatment of RA or do not plan on receiving follow-up care at the research centers

(6) Already a user of SSDM

4.3 Withdrawal criteria

Patients who request to withdraw from the clinical trial.

5. Study Design

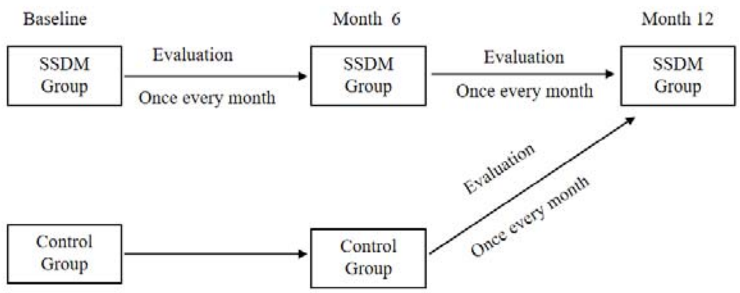

6. Statistical Procedures

6.1 Introduction

Mobile health applications (apps) have been shown to be effective in the management of chronic diseases with simple treatment target (e.g., hypertension). The potential clinical value of mobile health apps in RA has not been well studied.

The objective of this study is to investigate whether assessing patient reported outcomes using mobile health apps could result in improving outcomes in RA patients. This is a multicenter, randomized controlled trial in 22 tertiary hospitals across China. Eligible participants are adult RA patients with a smartphone. RA patients, regardless of baseline disease activity, will be included in this study. All patients will be followed up for 12-month. The statisticians and rheumatologists who assessed DAS28-CRP are blinded. Investigators and participants are not blind to group assignment. Participants will be randomly assigned at a 1:1 ratio (block size of four) to a smart system of disease management (SSDM) group or a conventional care control group. Upon the completion of 6-month parallel comparison, patients in the conventional care control group will be instructed to use the SSDM app for an extension of 6 months. The primary outcome is the rate of patients with DAS28-CRP ≤ 3.2 at month 6.

6.2 Clinical Study Site and Data Management

The study does not involve sample collection, storage sites and laboratory research sites.

The data in this study are divided into electronic data and paper data. The paper data should be archived and stored in the order of labeling for inspection. Electronic data files include databases, inspection procedures, analysis procedures, analysis results, code books and instruction documents, etc., which should be stored in categories. Multiple backups of electronic data should be stored on different disks or recording media to prevent damage. All original data should be kept within the required time limit.

6.3 Sample Size Determination

1) The rate of DAS28-CRP ≤ 3.2 at month 6 is assumed 44.3% in the control group and 52% in the SSDM group;[1-3] 2) 2-sided α at 0.05 and power at 90%; 3) 20% attrition rate. The calculation yields a total of 2200 patients. And 2204 is the minimum sample size created by the Random Number Generator.

6.4 Populations

(1) Intent-to-treat (ITT) population

The ITT population will include all randomized subjects. Patients will be analyzed according to the study arm to which they were randomized.

(2) Modified intent-to-treat (mITT) population

All participants who meet the inclusion criteria, do not meet the exclusion criteria and are randomized.

(3) Per-Protocol (PP) population

Patients who complete the treatment and follow-up and outcome information is available at each time point will be included in the PP population. The per protocol analysis includes only adherent participants (adherence rate > 50%). The number of patients in the PP analysis at month 6 was 968 in SSDM group and 886 in control group. The number of patients in the PP analysis at month 12 was 949 in SSDM group and 860 in control group.

Logistic regression, adjusting for baseline stratifications will be used to analyze the binary efficacy variables. The primary and second endpoints will be performed on both the mITT population and the PP population. The primary endpoint will also be analyzed using the worst-case scenario imputation, and the inverse probability of censoring weighted (IPCW) method.[4]

6.5 Efficacy Analysis

All efficacy analyses will be performed on both the mITT population and the PP population.

6.6 Primary endpoint analysis

The primary outcome is the rate of patients achieving DAS28-CRP (CRP) ≤ 3.2 at month 6. The categorical variables are described by frequency or percentage. We will report the differences between the two groups and associated 95% confidence intervals (CIs) using the Cochran-Mantel-Haenszel with adjustment for central effects. A nominal significance level of 0.05 (two-sided) will be applied to all the analyses. The statistical analyses and reporting will follow the CONSORT guidelines.

6.7 Major secondary analyses

The secondary endpoints include proportion of patients with moderate-to-good EULAR response rate at month 6 and month 12, ACR/EULAR Boolean remission rate month 6 and month 12, the change in simplified disease activity index (SDAI) month 6 and month 12, the change in clinical disease activity index (CDAI) month 6 and month 12, the change in tender joint count and swollen joint count month 6 and month 12, the change in Hospital Anxiety and Depression Scale (HADS) month 6 and month 12, the change in the 36-Item Short Form Survey (SF-36) month 6 and month 12, and flare rate at month 6 and month 12, the rate of patients with DAS28-CRP (CRP) ≤ 3.2 at month 12. The number and rate of adverse events. The normal distribution of the numeric variables will be examined using the Kolmogorov-Smirnov test. SDAI is continuously, while CDAI, swollen joint count, HADS and SF-36 are discrete variables. They will be used as continuous variables in this study. Descriptive data will be compared between the two arms using the student' s t test for normally distributed data and Wilcoxon rank-sum test for data that are not normally distributed.

For all categorical secondary endpoints, the differences between the two groups and associated 95% confidence intervals (CIs) will be calculated using the Cochran-Mantel-Haenszel with adjustment for center effects. For non-normal distributed continuous and discrete variables, the differences in medians and associated 95% CIs will be analyzed using quantile regression with adjustment for center effects. The median difference of outpatient visits between the two arms were calculated with the use of the Hodges-Lehmann estimated based on the Mann-Whitney U test.

6.8 Safety Analyses

This study does not involve safety issues, so safety evaluation is not required. The perceived adverse events will be collected. The adverse events were not assessed for both trial arms and were not able to be compared between the two groups.

Adverse events are defined as any general lack of well-being, symptoms of illness, or laboratory parameters outside the normal range.

Serious adverse events are defined as any life-threatening or fatal event.

6.9 Missing Data

The missing mechanism analysis will be performed. The missing data will be imputed using multiple imputation.

(1) Multiple-imputation method will be used to handle missing data under the missing at random assumptions.

(2) Five imputed data sets will be generated for the missing data. The missing values will be imputed using fully conditional specification (FCS) with the Multivariate Imputation by Chained Equations (mice) package (version 3.14.0) for R (version 4.2.1), and we will predictive mean matching (PMM) for the imputation of continuous variables. The baseline characteristics are included as auxiliary variables. Observed values and distributions of imputed data will be compared.

(3) The primary and secondary endpoints of each imputed datasets will be analyzed separately. And combined inferences from five imputed data sets are based on Rubin's rules.[5]

6.10 Other analyses plan

Pre-planned subgroup analyses will be conducted based on disease activity at baseline with adjustment by study centers, including
1) DAS 28-CRP ≤ 3.2 and DAS 28-CRP > 3.2;
2) DAS 28-CRP ≤ 3.2, 3.2-5.1, or > 5.1;
3) DAS 28-CRP ≤ 2.6, 2.6-3.2, 3.2-5.1, or > 5.1;

Other subgroup analyses will be post-hoc. No adjustments for multiple comparisons will be made for all post hoc analyses, which are considered exploratory.

References

1. Yang J, Wang Y, Li F, et al. Significant improvement of rheumatoid arthritis (RA) outcome with repeated self-assessment applying smart system of disease management (SSDM) mobiles tools: a cohort study of RA patients in china. *Ann Rheum Dis.* 2017;76(Suppl 2):1524. doi:http://dx.doi.org/10.1136/annrheumdis-2017-eular.5042

2. Liu JJ, Li R, Gan YZ, et al. Clinical deep remission and related factors in a large cohort of patients with rheumatoid arthritis. *Chin Med J (Engl)*. 2019;132(9):1009-1014. doi:10.1097/CM9.0000000000000227

3. Wang GY, Zhang SL, Wang XR, et al. Remission of rheumatoid arthritis and potential determinants: a national multi-center cross-sectional survey. *Clin Rheumatol*. 2015;34(2):221-30. doi:10.1007/s10067-014-2828-3

4. Adler AI, Latimer NR. Adjusting for Nonadherence or Stopping Treatments in Randomized Clinical Trials. JAMA. 2021;325(20):2110-2111. doi:10.1001/jama.2021.2433

5. Rubin DB. Multiple Imputation for Nonresponse in Surveys. New York: Wiley; 1987.

Appendix 1. Evaluation of Disease Activity

1. DAS28-CRP

DAS28 is calculated on the basis of CPR (DAS28-CRP).

$$DAS28\text{-}CRP = 0.56 * \text{Sqrt}(TJC28) + 0.28 * \text{Sqrt}(SJC28) + 0.36 * \ln(CRP + 1) + 0.014 * GH + 0.96$$

Sqrt: square root

TJC: 28 Tender joint count

SJC: 28 Swollen joint count

CRP: C-reactive protein

GH: General Health on a 100mm. Visual Analogue Scale.

Interpretation

Remission (REM) ≤ 2.6;

Low Disease Activity (LDA) 2.6-3.2;

Moderate Disease Activity (MDA) 3.2-5.1;

High Disease Activity (HDA) > 5.1

2. EULAR response criteria

	DAS improvement		
DAS28-CRP at endpoint	> 1.2	0.6-1.2	≤ 0.6
≤ 3.2	Good response	Moderate response	No response
3.2 to ≤ 5.1	Moderate response	Moderate response	No response
> 5.1	Moderate response	No response	No response

3. ACR/EULAR Boolean remission

At any time point, patient must satisfy all of the following:

(1) Swollen joint count (SJC) ≤ 1

(2) Tender joint count (TJC) ≤ 1

(3) Patient Global assessment ≤ 1 (on a 0-10 scale)

(4) CRP ≤ 1mg/dL

4. Clinical Disease Activity Index (CDAI)

The CDAI is calculated as follows:

CDAI = SJC28 + TCJ28 + PtGA + PhGA

SJC = Swollen Joint Count

TJC = Tender Joint Count

PtGA = Patient Global Assessment of Disease Activity

PhGA = Physician Global Assessment of Disease Activity

Interpretation

Remission ≤ 2.8;

Low Disease Activity 2.9-10.0;

Moderate Disease Activity 10.1-22.0;

High Disease Activity > 22.0

5. Simple Disease Activity Index (SDAI)

The SDAI is a tool for measurement of disease activity in RA that integrates measures of physical examination, acute phase response, patient self-assessment, and evaluator assessment.

The SDAI is calculated as follows:

SDAI = SJC28 + TCJ28 + PtGA + PhGA + CRP

SJC = Swollen Joint Count (0 to 28)

TJC = Tender Joint Count (0 to 28)

PtGA = Patient Global Assessment of Disease Activity (VAS; 0 to 10 cm)

PhGA = Physician Global Assessment of Disease Activity (VAS; 0 to 10 cm)

CRP = C-reactive protein in mg/dL

Interpretation

Remission ≤ 3.3;

Low Disease Activity 3.4-11.0;

Moderate Disease Activity 11.1-26.0;

High disease activity > 26.0

6. Patient's global assessment of disease activity (PtGA)

The patient’s global assessment of disease activity (PtGA) is used to assess the patient reporting disease activity. The patient will be asked to assess their disease during the past week on a visual analogue scale (VAS; 0 to 10 cm). The anchors of the instrument include 0 to represent ‘the best’ and 10 to represent ‘the worst’.

7. Physician's global assessment of disease activity (PhGA)

The physician’s global assessment of disease activity (PhGA) is used to assess the physician evaluating disease activity. The physician will assess a patient's disease activity during the past week on a visual analogue scale (VAS; 0 to 10 cm). The anchors of the instrument include 0 to represent ‘the best’ and 10 to represent ‘the worst’.

Appendix 2. 2010 ACR/EULAR RA classification criteria

Domain	Category	Point score
A	Joint involvement (0-5 points) [a]	
	1 large joint	0
	2-10 large joints	1
	1-3 small joints (large joints not counted)	2
	4-10 small joints (large joints not counted)	3
	> 10 joints including at least one small joint	5
B	Serology (at least one test needed for classification; 0-3 points)[b]	
	Negative RF and negative ACPA	0
	Low positive RF or low positive ACPA	2
	High positive RF or high positive ACPA	3
C	Acute-phase reactants (at least one test needed for classification; 0-1 point) [c]	
	Normal CRP and normal ESR	0
	Abnormal CRP or abnormal ESR	1
D	Duration of symptoms [d]	
	< 6 weeks	0
	≥ 6 weeks	1

The criteria are meant to be applied in patients with at least one swollen joint, after the exclusion of other causes of synovitis. The points from each of domain A through D are added and the sum is considered to be the total score. A total score of ≥ 6 is needed to classify a patient as having definite RA.

[a] Joint involvement refers to any swollen or tender joint on examination, which may be confirmed by imaging evidence of synovitis. DIP joints, first CMC joints and first MTP joints are excluded from assessment. Large joints refer to shoulders, elbows, hips, knees and ankles. Small joints refer to MCP joints, PIP joints, second through fifth MTP joints, thumb IP joints and wrists.

[b] Negative means less than or equal to the upper limit of normal (ULN); low positive means > ULN; high positive means > 3 × ULN.

[c] Normal and abnormal are determined by local laboratory standards.

[d] Duration of symptoms as per patient’ s self-report.

Appendix 3. 36-Item Short Form Survey Instrument (SF-36)

Choose one option for each questionnaire item.

1. In general, would you say your health is:
① Excellent ② Very good ③ Good ④ Fair ⑤ Poor

2. Compared to one year ago, how would you rate your health in general now?
(1) Much better now than one year ago
(2) Somewhat better now than one year ago
(3) About the same
(4) Somewhat worse now than one year ago
(5) Much worse now than one year ago
(The score is 1, 2, 3, 4, 5 for ①-⑤)

3. The following items are about activities you might do during a typical day. Does your health now limit you in these activities? If so, how much?
(1) Vigorous activities, such as running, lifting heavy objects, participating in strenuous sports
① Yes, limited a lot ② Yes, limited a little ③ No, not limited at all
(The score is 1, 2, 3 for ①-③the same below)
(2) Moderate activities, such as moving a table, pushing a vacuum cleaner, bowling, or playing golf
① Yes, limited a lot ② Yes, limited a little ③ No, not limited at all
(3) Lifting or carrying groceries
① Yes, limited a lot ② Yes, limited a little ③ No, not limited at all
(4) Climbing several flights of stairs
① Yes, limited a lot ② Yes, limited a little ③ No, not limited at all
(5) Climbing one flight of stairs
① Yes, limited a lot ② Yes, limited a little ③ No, not limited at all
(6) Bending, kneeling, or stooping
① Yes, limited a lot ② Yes, limited a little ③ No, not limited at all
(7) Walking more than a mile
① Yes, limited a lot ② Yes, limited a little ③ No, not limited at all
(8) Walking several blocks
① Yes, limited a lot ② Yes, limited a little ③ No, not limited at all
(9) Walking one block
① Yes, limited a lot ② Yes, limited a little ③ No, not limited at all
(10) Bathing or dressing yourself
① Yes, limited a lot ② Yes, limited a little ③ No, not limited at all

4. During the past 4 weeks, have you had any of the following problems with your work or other regular daily activities as a result of your physical health?
(1) Cut down the amount of time you spent on work or other activities
① Yes ② No
(The score is 1, 2 for ①-② the same below)
(2) Accomplished less than you would like
① Yes ② No
(3) Were limited in the kind of work or other activities
① Yes ② No
(4) Had difficulty performing the work or other activities (for example, it took extra effort)
① Yes ② No

5. During the past 4 weeks, have you had any of the following problems with your work or other regular daily activities as a result of any emotional problems (such as feeling depressed or anxious)?
(1) Cut down the amount of time you spent on work or other activities
① Yes ② No
(The score is 1, 2 for ①-② the same below)
(2) Accomplished less than you would like
① Yes ② No
(3) Didn't do work or other activities as carefully as usual
① Yes ② No

6. During the past 4 weeks, to what extent has your physical health or emotional problems interfered with your normal social activities with family, friends, neighbors, or groups?
① Not at all ② Slightly ③ Moderately ④ Quite a bit ⑤ Extremely

(The score is 5, 4, 3, 2, 1 for ①-⑤)

7. How much bodily pain have you had during the past 4 weeks?

① None ② Very mild ③ Mild ④ Moderate ⑤ Severe ⑥ Very severe

(The score is 6, 5.4, 4.2, 3.1, 2.2, 1 for ①-⑤)

8. During the past 4 weeks, how much did pain interfere with your normal work (including both work outside the home and housework)?

① Not at all ② A little bit ③ Moderately ④ Quite a bit ⑤ Extremely

(If neither 7 nor 8 is yes, the score is 6, 4.75, 3.5, 2.25, 1.0 for ①-⑤; if 7 is yes and 8 is no, the score is 5, 4, 3, 2, 1 for ①-⑤)

9. These questions are about how you feel and how things have been with you during the past 4 weeks. For each question, please give the one answer that comes closest to the way you have been feeling.

How much of the time during the past 4 weeks...

(1) Did you feel full of pep?

① All of the time ② Most of the time ③ A good bit of the time ④ Some of the time ⑤ A little of the time ⑥ None of the time

(The score is 6, 5, 4, 3, 2, 1 for ①-⑥)

(2) Have you been a very nervous person?

① All of the time ② Most of the time ③ A good bit of the time ④ Some of the time ⑤ A little of the time ⑥ None of the time

(The score is 1, 2, 3, 4, 5, 6 for ①-⑥)

(3) Have you felt so down in the dumps that nothing could cheer you up?

① All of the time ② Most of the time ③ A good bit of the time ④ Some of the time ⑤ A little of the time ⑥ None of the time

(The score is 1, 2, 3, 4, 5, 6 for ①-⑥)

(4) Have you felt calm and peaceful?

① All of the time ② Most of the time ③ A good bit of the time ④ Some of the time ⑤ A little of the time ⑥ None of the time

(The score is 6, 5, 4, 3, 2, 1 for ①-⑥)

(5) Did you have a lot of energy?

① All of the time ② Most of the time ③ A good bit of the time ④ Some of the time ⑤ A little of the time ⑥ None of the time

(The score is 6, 5, 4, 3, 2, 1 for ①-⑥)

(6) Have you felt downhearted and blue?

① All of the time ② Most of the time ③ A good bit of the time ④ Some of the time ⑤ A little of the time ⑥ None of the time

(7) Did you feel worn out?

① All of the time ② Most of the time ③ A good bit of the time ④ Some of the time ⑤ A little of the time ⑥ None of the time

(The score is 1, 2, 3, 4, 5, 6 for ①-⑥)

(8) Have you been a happy person?

① All of the time ② Most of the time ③ A good bit of the time ④ Some of the time ⑤ A little of the time ⑥ None of the time

(The score is 6, 5, 4, 3, 2, 1 for ①-⑥)

(9) Did you feel tired?

① All of the time ② Most of the time ③ A good bit of the time ④ Some of the time ⑤ A little of the time ⑥ None of the time

(The score is 1, 2, 3, 4, 5, 6 for ①-⑥)

10. During the past 4 weeks, how much of the time has your physical health or emotional problems interfered with your social activities (like visiting with friends, relatives, etc.)?

① All of the time ② Most of the time ③ A good bit of the time ④ Some of the time ⑤ A little of the time ⑥ None of the time

(The score is 1, 2, 3, 4, 5, 6 for ①-⑥)

11. How TRUE or FALSE is each of the following statements for you.

(1) I seem to get sick a little easier than other people

① Definitely true ② Mostly true ③ Don't know ④ Mostly false ⑤ Definitely false

(The score is 1, 2, 3, 4, 5 for ①-⑤)

(2) I am as healthy as anybody I know

① Definitely true ② Mostly true ③ Don't know ④ Mostly false ⑤ Definitely false
(The score is 5, 4, 3, 2, 1 for ①-⑤)
(3) I expect my health to get worse
① Definitely true ② Mostly true ③ Don't know ④ Mostly false ⑤ Definitely false
(The score is 1, 2, 3, 4, 5 for ①-⑤)
(4) My health is excellent
① Definitely true ② Mostly true ③ Don't know ④ Mostly false ⑤ Definitely false
(The score is 5, 4, 3, 2, 1 for ①-⑤)

SF-36 score calculation

Item	Score	Item	Score	Item	Score	Item	Score
1		3-(9)		7		9-(9)	
2		3-(10)		8		10	
3-(1)		4-(1)		9-(1)		11-(1)	
3-(2)		4-(2)		9-(2)		11-(2)	
3-(3)		4-(3)		9-(3)		11-(3)	
3-(4)		4-(4)		9-(4)		11-(4)	
3-(5)		5-(1)		9-(5)			
3-(6)		5-(2)		9-(6)			
3-(7)		5-(3)		9-(7)			
3-(8)		6		9-(8)		Sum	

Appendix 4. The modified Health Assessment Questionnaire Disability index

Please check (√) the one best answer for your abilities over the past week:

Over the past week were you able to	Without any difficulty	With some difficulty	With much difficulty	Unable to do
Dressing & grooming				
Dress yourself, including tying shoelaces and doing buttons?				
Arising				
Get in and out of bed?				
Eating				
Lift a full cup or glass to your mouth?				
Walking				
Walk outdoors on flat ground?				
Hygiene				
Wash and dry your entire body?				
Reach				
Bend down and pick up clothing from the floor?				
Grip				
Turn faucets on and off?				
Activities				
Get in and out of a car?				
