# Supplementary material for: Management of Rheumatoid Arthritis With a Digital Health Application: A Multicenter, Pragmatic Randomized Clinical Trial
Source: JAMA Netw Open. 2023 Apr 14;6(4):e238343. doi: 10.1001/jamanetworkopen.2023.8343 (PMC10105314; doi:10.1001/jamanetworkopen.2023.8343)
Supplement: Supplement 2. — eTable 1. Study Inclusion and Exclusion Criteria eMethods. eTable 2. Subgroup Analysis of Patients With DAS28-CRP ≤ 3.2 at Month 6 in the Per-Protocol Analysis eTable 3. Subgroup Analysis of Patients With DAS28-CRP > 3.2 at Month 6 in the Per-Protocol Analysis eTable 4. Comparison of Outcomes at Month 6 and Month 12 in Patients With or Without Intervention Upon Alert From SSDM eTable 5. The Change in Treatment Between Patients With or Without Intervention eTable 6. The Rate of DAS28-CRP ≤ 3.2 Among Participants With Alerts and Intervention eTable 7. Safety Data From Baseline to Month 6 eFigure. The Rate of Patients With DAS28-CRP ≤ 3.2 at Month 6 [file jamanetwopen-e238343-s002.pdf]

Supplemental Online Content

Li C, Huang J, Wu H, et al. Management of rheumatoid arthritis with a digital health application: a multicenter, pragmatic randomized clinical trial. *JAMA Netw Open*. 2023;6(4):e238343. doi:10.1001/jamanetworkopen.2023.8343

eTable 1. Study Inclusion and Exclusion Criteria
eMethods.
eTable 2. Subgroup Analysis of Patients With DAS28-CRP ≤ 3.2 at Month 6 in the Per-Protocol Analysis
eTable 3. Subgroup Analysis of Patients With DAS28-CRP > 3.2 at Month 6 in the Per-Protocol Analysis
eTable 4. Comparison of Outcomes at Month 6 and Month 12 in Patients With or Without Intervention Upon Alert From SSDM
eTable 5. The Change in Treatment Between Patients With or Without Intervention
eTable 6. The Rate of DAS28-CRP ≤ 3.2 Among Participants With Alerts and Intervention
eTable 7. Safety Data From Baseline to Month 6
eFigure. The Rate of Patients With DAS28-CRP ≤ 3.2 at Month 6

This supplemental material has been provided by the authors to give readers additional information about their work.

© 2023 Li C et al. *JAMA Network Open.*

eTable 1. Study Inclusion and Exclusion Criteria

**Inclusion Criteria**
1) Diagnosis of RA by investigators according to the 2010 American College of Rheumatology (ACR)/European League Against Rheumatism (EULAR) criteria
2) ≥18 years old
3) Have a smartphone
4) Able to evaluate disease activity by themselves
**Exclusion Criteria**
1) Not able to read and use smartphone
2) Not able to perform the joint examination by themselves
3) Patients with cognitive impairment or mental condition which make them unable to comply with the requirements of self-management
4) Hand deformations or any other conditions (e.g., Parkinson's disease and late-stage Alzheimer's disease) that affect data input
5) Do not want to accept the treatment of RA or do not plan on receiving follow-up care at the research centers
6) Already a user of SSDM

© 2023 Li C et al. *JAMA Network Open.*

eMethods.

1. Supplemental Methods

(1) Screening procedure

Outpatient and inpatient RA patients in the study centers during routine medical visits will be informed about the study by their attending rheumatologists. Potentially eligible participants who are suitable and interested in this study will be given the study information sheet, which will allow them to obtain a comprehensive view of the study. If consent is given, the research investigators will be introduced to the potentially eligible participants.

The investigators will explain the trial to them, check inclusion and exclusion criteria, and tell the patients that they are free to discontinue from the study at any time. The investigator will also discuss the importance of participation in the study with the potentially eligible participants. The potentially eligible participants will be given opportunities to ask questions and will be allowed adequate time for consideration. If the potentially eligible participants have a CRP performed as part of their routine lab tests within 2 weeks, the result will be used for the study. No additional lab tests are needed. If the potentially eligible participants do not have a CRP, CRP with other routine lab tests will be performed in these patients. The potentially eligible participants will return to the hospital within two weeks.

Potentially eligible participants should sign the informed consent. Following informed consent, the evaluator will assess the participants' DAS28-CRP.

Following the evaluation by rheumatologists, eligible participants will be randomized by an interactive web response system at a 1:1 ratio to a SSDM arm vs a conventional care control arm.

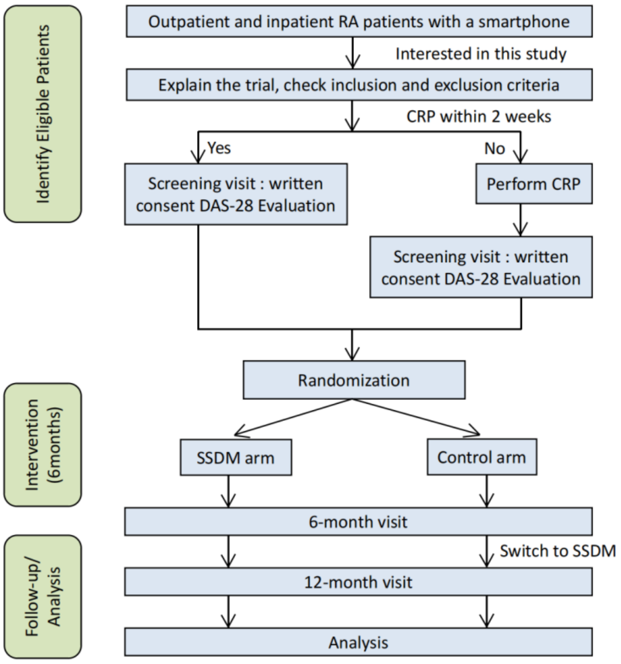

Eligibility, enrollment, randomization and follow-up of the study

Patients randomized to the SSDM arm will watch a 15-minute video that described the key features of SSDM to allow correct use of the app. After learning, the patients will perform the self-assessment all by themselves using the SSDM. If there are inconsistencies between the patient and physician, the physician will correct it, and then the patient will do it again. Finally, physicians will confirm that the patient could

© 2023 Li C et al. *JAMA Network Open.*

perform the evaluation of DAS28-CRP using SSDM all by themselves.

(2) Quality Control

1) All investigators and research staff at each site received training on the protocol of this study. They were asked to keep neutral and not allowed to recommend the app to patients in the control arm. We obtained all the telephone numbers and IDs of patients in the control arm, and they could not register during the first 6 months of the study.

2) The rheumatologists who assessed the disease activity at baseline, month 6 and month 12 were independent of the rest of the study team. They received a training on joint assessment before the study began. They performed swollen and tender joint counts for all study visits whenever possible.

(3) Adherence improvement

Adherence was encouraged by smartphone notifications, or by investigator via telephone during the study period. Three days before the evaluation, smartphone notifications would be sent to the participants. Two days before the evaluation date, short messages would be sent to the participants. If the patients did not complete the evaluation 1 day after the evaluation day, the investigator would make a telephone call to the patients to remind the patients to finish the evaluation.

Adherence was defined as the ratio of actual self-assessment numbers against the required self-assessment numbers.

(4) Intervention upon alert

After uploading the patient-reported outcomes and the lab by the participants, the SSDM uploaded and synchronized the results to the rheumatologist's interface. The assigned rheumatologists could monitor the alert online, and instruct the patients to come back for outpatient visits or refill or make new prescriptions. And a clear instruction was recorded by the rheumatologists. If there was no action from the rheumatologist, the research staff reminded the rheumatologist at the same time. No instruction was made if the rheumatologist did not response to alert or the patient could not come back for an outpatient visit. The research staff reviewed the alert and remind the rheumatologist every day.

The use of conventional synthetic (cs) disease-modifying antirheumatic drugs (DMARDs), glucocorticoids (GCs), biological (b) DMARDs, targeted synthetic (ts) DMARDs and non-steroidal anti-inflammatory drugs (NSAIDs) was recorded in the SSDM.

The definition of medication change was the addition of any new DMARDs or GCs, the withdrawal of any DMARDs or GCs, an increase in the dose of any DMARDs or GCs, or a decrease in the dose of a DMARDs or GCs.

The definition of dose change was the adjustment or modification of the amount or frequency of DMARDs and/or GCs.

(5) Laboratory Parameters

The laboratory test could be based on the report by any of the medical institutions that the patients opted to visit.

(6) Adverse events reporting

None of the adverse events were related to the intervention of mobile health apps. We collected adverse events reported by both patients and rheumatologists.

1) Patient-reported: The symptoms of adverse events were mainly reported by patients following a systematic assessment of a menu of possible adverse events.

2) Reported by the study rheumatologists: The study rheumatologists could also report the adverse events from the doctors' side.

Comparisons between the SSDM arm and control arm were not performed.

(7) Standardization of SF-36

The physical component score (PCS) and mental component score (MCS) of the SF-36 are scored in three steps.

1) Standardize each of the 8 SF-36 scales using a z-score transformation. The mean and standard deviation are from the general population.[1]

2) Compute the aggregate score for the PCS and MCS by using the physical and mental factor score coefficients from the general population.[1]

3) Transform each component score to the norm-based (50, 10) scoring.

(8) Missing data

1) The missing mechanism of the missing data

Baseline DAS28-CRP, sex, age, group, and 6-month DAS28-CRP were included in two-sample *t*-test, and expectation-maximization algorithm (EM) to identify whether the 6-month DAS28-CRP was missing completely at random (MCAR). Group was coded as a dummy variable, with value 1 for SSDM arm, and 0 for control arm. The number and proportion of missing 6-month DAS28-CRP were 343 and 15.6% (343/2197), respectively. There was no missing value for baseline DAS28-CRP, age, group, or sex. The mean baseline DAS28-CRP among patients with and without missing data were 4.05 and 3.73, respectively. Moreover, the EM algorithm identified that the correlation coefficient between baseline and 6-month DAS28-CRP was 0.462. Meanwhile, Little's MCAR test implied that the missing mechanism of 6-month DAS28-CRP was not MCAR ($P < .05$).

A further logistic regression model was used to indicate whether the missing mechanism of 6-month DAS28-CRP was missing at random (MAR). The missing value of 6-month DAS28-CRP was labeled as Y = 1, and the non-missing value of DAS28-CRP was labeled as Y = 0; Y was the dependent variable in the logistic regression, and the independent variables were baseline DAS28-CRP, sex, age, and group. Logistic regression model results showed that the missing probability of 6-month DAS28-CRP was not significantly related to sex ($P = .52$) and age ($P = .53$), and was significantly related to group ($P < .001$) and baseline DAS28-CRP ($P < .001$), indicating that the missing mechanism of 6-month DAS28-CRP was MAR.

2) Multiple imputation

① The missing data were imputed using multiple imputation in the condition of MAR.

② Five imputed data sets were generated for the missing data. The missing values were imputed using fully conditional specification (FCS) with the Multivariate Imputation by Chained Equations (mice) package (version 3.14.0) for R (version 4.2.1), and we used predictive mean matching (PMM) for the imputation of continuous variables. The baseline characteristics were included as auxiliary variables. Observed values and distributions of imputed data were compared.

③ The primary and secondary endpoints of each imputed datasets were analyzed separately. And combined inferences from five imputed data sets are based on Rubin's rules[2]. All primary and secondary endpoints analyses were adjusted for center effect using the Cochran-Mantel-Haenszel or quantile regression.

2. The characteristics of the study sites

There were 22 tertiary hospitals from 16 cities in China. Eleven hospitals were located in the south of China, and 11 hospitals were located in the north of China. Out of these 22 hospitals, 19 were university-affiliated hospitals, and 3 hospitals were not university-affiliated hospitals. The characteristics of the study centers were shown in eTable 1. The median number of investigators is 2

© 2023 Li C et al. *JAMA Network Open.*

(IQR 2-2). The median numbers of participants was 47 (IQR 38 - 56) in the SSDM arm and 49 (IQR 34 - 65) in the control arm.

The characteristics of the study sites

	Hospital	Location	Grade	Levels	No. Of investigator	No.of patients
1	Peking University People’s Hospital	Beijing	A	Tertiary	4	195
2	The Sixth Affiliated Hospital of Sun Yat-sen University	Guangzhou	A	Tertiary	2	169
3	The Second Affiliated Hospital of Zhejiang University School of Medicine	Hangzhou	A	Tertiary	2	115
4	The Second Xiangya Hospital of Central South University	Changsha	A	Tertiary	2	146
5	Xuanwu Hospital Capital Medical University	Beijing	A	Tertiary	2	103
6	Linyi People’s Hospital	Linyi	A	Tertiary	2	102
7	Peking University International Hospital	Beijing	/	Tertiary	2	105
8	Northern Jiangsu People's Hospital	Yangzhou	A	Tertiary	2	71
9	The First Affiliated Hospital of Nanjing Medical University	Nanjing	A	Tertiary	2	19
10	Shandong Provincial Hospital Affiliated to Shandong First Medical University	Jinan	A	Tertiary	3	115
11	Mianyang Central Hospital	Mianyang	A	Tertiary	3	108
12	The First People's Hospital of Yunnan Province	Kunming	A	Tertiary	3	111
13	The First Affiliated Hospital of USTC	Hefei	A	Tertiary	2	120
14	Tianjin First Central Hospital	Tianjin	A	Tertiary	2	76
15	Tianjin Medical University General Hospital	Tianjin	A	Tertiary	2	113
16	Zhejiang Provincial People’s Hospital	Hangzhou	A	Tertiary	2	75
17	Bethune International Peace Hospital	Shijiazhuang	A	Tertiary	2	86
18	The First Affiliated Hospital of Baotou Medical College	Baotou	A	Tertiary	3	48
19	Hebei General Hospital	Shijiazhuang	A	Tertiary	2	72
20	Tungwah Hospital of Sun Yat-sen University	Dongguan	A	Tertiary	2	74
21	The First Affiliated Hospital of Anhui Medical University	Hefei	A	Tertiary	2	105
22	Xijing Hospital	Xi'an	A	Tertiary	2	69

eReferences

1. Ware JE, Kosinski M, Dewey JE. *How to Score Version 2 of the Sf-36 Health Survey (Standard & Acute Forms)*. Lincoln R.I:QualityMetric;2000.
2. White IR, Royston P, Wood AM. Multiple imputation using chained equations: Issues and guidance for practice. *Stat Med*. 2011;30(4):377-399. doi: 10.1002/sim.4067.

eTable 2. Subgroup Analysis of Patients With DAS28-CRP ≤ 3.2 at Month 6 in the Per-Protocol Analysis

	**SSDM (n=968)**	**Control (n=886)**	**Group difference (95%CI)**	***P* value**
Baseline DAS28-CRP				
DAS28-CRP ≤ 3.2, n (%)	326 (89.8)	292 (85.1)	4.8% (0 to 9.6%)	.06
DAS28-CRP > 3.2, n (%)	370 (61.2)	285 (52.5)	8.3% (2.7% to 13.8%)	.003
Baseline DAS28-CRP				
DAS28-CRP ≤ 3.2, n (%)	326 (89.8)	292 (85.1)	4.8% (0 to 9.6%)	.06
DAS28-CRP 3.2 - 5.1, n (%)	283 (64.9)	217 (55.8)	8.1% (1.5% to 14.6%)	.02
DAS28-CRP > 5.1, n (%)	87 (51.5)	68 (44.2)	9.6% (-0.8% to 19.9%)	.07
Baseline DAS28-CRP				
DAS28-CRP ≤ 2.6, n (%)	203 (90.2)	192 (89.7)	-1.0% (-6.4% to 4.5%)	.74
DAS28-CRP 2.6 - 3.2, n (%)	123 (89.1)	100 (77.5)	13.4% (4.3% to 22.5%)	.004
DAS28-CRP 3.2 - 5.1, n (%)	283 (64.9)	217 (55.8)	8.1% (1.5% to 14.6%)	.02
DAS28-CRP > 5.1, n (%)	87 (51.5)	68 (44.2)	9.6% (-0.8% to 19.9%)	.07

© 2023 Li C et al. *JAMA Network Open.*

eTable 3. Subgroup Analysis of Patients With DAS28-CRP > 3.2 at Month 6 in the Per-Protocol Analysis

	SSDM (n=968)	Control (n=886)	Group difference (95%CI)	*P* value
Baseline DAS28-CRP				
DAS28-CRP ≤ 3.2, n (%)	37 (10.2)	51 (14.9)	-4.8% (-9.6% to 0)	.06
DAS28-CRP > 3.2, n (%)	235 (38.8)	258 (47.5)	-8.3% (-13.8% to -2.7%)	.003
Baseline DAS28-CRP				
DAS28-CRP ≤ 3.2, n (%)	37 (10.2)	51 (14.9)	-4.8% (-9.6% to 0)	.06
DAS28-CRP 3.2-5.1, n (%)	153 (35.1)	172 (44.2)	-8.1% (-14.6% to -1.5%)	.02
DAS28-CRP > 5.1, n (%)	82 (48.5)	86 (55.8)	-9.6% (-19.9% to 0.8%)	.07
Baseline DAS28-CRP				
DAS28-CRP ≤ 2.6, n (%)	22 (9.8)	22 (10.3)	0.9% (-4.5% to 6.4%)	.74
DAS28-CRP 2.6-3.2, n (%)	15 (10.9)	29 (22.5)	-13.4% (-22.5% to -4.3%)	.004
DAS28-CRP 3.2-5.1, n (%)	153 (35.1)	172 (44.2)	-8.1% (-14.6% to -1.5%)	.02
DAS28-CRP > 5.1, n (%)	82 (48.5)	86 (55.8)	-9.6% (-19.9% to 0.8%)	.07

© 2023 Li C et al. *JAMA Network Open.*

7 **eTable 4. Comparison of Outcomes at Month 6 and Month 12 in Patients With or Without Intervention Upon Alert From SSDM**

	Month 6				Month 12			
	**Intervention (n=78)**	**Non-Intervention (n=124)**	**Group difference (95% CI)**	***P* value**	**Intervention (n=193)**	**Non-Intervention (n=796)**	**Group difference (95% CI)**	***P* value**
DAS28-CRP, median (IQR)	2.8 (2.1 to 3.2)	2.8 (2.3 to 3.8)	0 (-0.3 to 0.2)	.78	2.4 (1.8 to 3.0)	3.0 (2.2 to 3.6)	-0.6 (-0.8 to -0.4)	<.001
DAS28-CRP ≤ 3.2, n (%)	60 (76.9)	79 (63.7)	13.2% (0.6% to 25.8%)	.048	160 (82.9)	445 (55.9)	27.0% (20.3% to 33.3%)	<.001
Moderate to good EULAR response, n (%)	58 (74.4)	74 (59.7)	14.7% (1.7% to 27.7%)	.03	152 (78.8)	515 (64.7)	14.6% (8.0% to 21.1%)	<.001
ACR/EULAR Boolean remission, n (%)	7 (9.0)	7 (5.7)	3.3% (-4.2% to 10.9%)	.36	27 (14.0)	86 (10.8)	3.2% (-2.2% to 8.5%)	.21
CDAI, median (IQR)	8.3 (5.9 to 11.5)	9.5 (6.0 to 14.7)	-1.1 (-2.9 to 0.7)	.23	5.9 (4.0 to 9.3)	9.0 (5.5 to 12.0)	-3.1 (-3.6 to -2.6)	<.001
SDAI, median (IQR)	9.0 (6.8 to 13.0)	10.2 (7.2 to 15.9)	-0.9 (-3.0 to 1.2)	.39	6.6 (4.4 to 9.8)	10.0 (6.4 to 13.8)	-3.5 (-4.2 to -2.7)	<.001
Tender joint counts, median (IQR)	1 (0 to 2)	1.5 (0 to 3)	0 (-1 to 1)	>.99	1 (0 to 2)	2 (0 to 3)	-1 (-2 to 0)	.04
Swollen joint counts, median (IQR)	0 (0 to 1)	0 (0 to 2)	0 (0 to 0)	>.99	0 (0 to 0)	0 (0 to 2)	0 (0 to 0)	>.99
PtGA , median (IQR)	31.0 (20.0 to 50.0)	37.0 (21.5 to 50.0)	-6.0 (-13.9 to 1.9)	.14	22.0 (13.0 to 34.0)	30.0 (16.0 to 49.0)	-8.0 (-11.4 to -4.6)	<.001
PhGA, median (IQR)	29.0 (20.0 to 50.0)	37.0 (22.2 to 50.0)	-8.0 (-17.1 to 1.1)	.09	22.0 (12.0 to 34.0)	27.0 (13.0 to 43.0)	-5.0 (-9.0 to -1.1)	.01
SF-36 PCS, median (IQR)	49.4 (42.7 to 54.2)	49.8 (42.4 to 56.0)	-0.2 (-4.6 to 4.2)	.93	56.2 (42.2 to 59.3)	56.4 (49.8 to 60.1)	0.2 (-3.8 to 4.2)	.93

© 2023 Li C et al. *JAMA Network Open.*

	Month 6				Month 12			
	**Intervention (n=78)**	**Non-Intervention(n =124)**	**Group difference (95% CI)**	***P* value**	**Intervention (n=193)**	**Non-Intervention(n=796)**	**Group difference (95% CI)**	***P* value**
SF-36 MCS, median (IQR)	36.8 (30.1 to 41.6)	36.9 (32.7 to 42.2)	-0.4 (-3.9 to 4.7)	.85	38.4 (35.1 to 40.6)	38.9 (34.9 to 42.7)	-0.4(-2.3 to 1.6)	.71
HADS, median (IQR)								
-A	5.0 (2.0 to 7.0)	7.0 (4.0 to 8.0)	-2.0 (-3.7 to -0.3)	.02	6.0 (3.0 to 7.0)	2.0 (0 to 6.0)	4.0 (2.3 to 5.7)	<.001
-D	6.0 (3.0 to 8.0)	6.0 (3.0 to 9.0)	0 (-1.4 to 1.4)	>.99	6.0 (2.0 to 7.0)	2.0 (0 to 6.0)	4.0 (1.7 to 6.1)	<.001
mHAQ score, median (IQR)	0 (0 to 2)	0.5 (0 to 3)	0 (-1.3 to 1.3)	>.99	0 (0 to 2)	0 (0 to 1)	0 (0 to 0)	>.99

Data are n (%) or median (IQR). Differences are median differences or differences between proportions

Abbreviations: DAS28-CRP, 28-joint disease activity score using C-reactive protein. EULAR, European League Against Rheumatism. ACR, American College of Rheumatology. CDAI, Clinical Disease Activity Index. SDAI, Simplified Disease Activity Index. PtGA, Patient’s global assessment of disease activity. PhGA, Physician global assessment of disease activity. SF-36, the 36-Item Short Form Survey. PCS, physical component score. MCS, mental component score. HADS, Hospital Anxiety and Depression Scale. mHAQ, modified Health Assessment Questionnaire.

© 2023 Li C et al. *JAMA Network Open.*

eTable 5. The Change in Treatment Between Patients With or Without Intervention

	Month 6				Month 12			
	**Intervention (n=78)**	**Non-Intervention (n=124)**	**Group difference (95% CI)**	***P* value**	**Intervention (n=193)**	**Non-Intervention (n=796)**	**Group difference (95% CI)**	***P* value**
Medication changes, n (%)	23 (29.5)	42 (33.9)	-4.4% (-16.9% to 9.0%)	.52	73 (37.8)	204 (25.6)	12.2% (4.9% to 19.8%)	<.001
Dose change, n (%)	7 (9.0)	0 (0.0)	9.0% (2.7% to 18.2%)	.001	19 (9.8)	0 (0.0)	9.8% (6.1% to 15.2%)	<.001
Treatment of flare, n (%)	11 (14.1)	13 (10.5)	3.6% (-5.4% to 14.0%)	.44	37 (19.2)	146 (18.3)	0.9% (-4.9% to 7.5%)	.79
Use of steroid[a], n(%)	5 (6.4)	12 (9.7)	-3.3% (-10.7% to 5.5%)	.42	10 (5.2)	32 (4.0)	1.2% (-1.6% to 5.4%)	.47
Use of NSAIDs[a], n(%)	9 (11.5)	14 (11.3)	0.2% (-8.4% to 10.2%)	.96	14 (7.3)	69 (8.7)	-1.4% (-5.0% to 3.5%)	.52
Outpatient visits, n (%)	52 (66.7)	88 (71.0)	-4.3% (-17.5% to 8.4%)	.52	120 (62.2)	466 (58.5)	3.6% (-4.2% to 11.0%)	.36

[a]add-on of steroid or NSAIDs

Abbreviation: Nonsteroidal Anti-inflammatory Drugs

© 2023 Li C et al. *JAMA Network Open.*

eTable 6. The Rate of DAS28-CRP ≤ 3.2 Among Participants With Alerts and Intervention

		**Numbers of Alerts**						**Risk ratio (95% CI)**	***P* value**
			**≥ 2**						
		**1**	**2**	**3**	**4**	**5**	**Total**		
Month 6	Numbers of patients with alert, n	180	20	2			22		
	Numbers of patients with intervention, n	72	6	0			6		
	DAS28 ≤ 3.2 among patients with alert, n (%)	125 (69.4)	14 (70.0)	0 (0)			14 (63.6)	0.916 (0.659 to 1.275)	.58[a]
	DAS28 ≤ 3.2 among patients with intervention, n (%)	54 (75.0)	6 (100)	0			6 (100)	1.333 (1.167 to 1.524)	.33[b]
Month 12	Numbers of patients with alert, n	784	162	34	8	1	205		
	Numbers of patients with intervention, n (%)	131 (16.7)	50 (30.8)	10 (29.4)	2 (25.0)	0 (0)	62		
	DAS28 ≤ 3.2 among patients with alert, n (%)	493 (62.9)	89 (54.9)	19 (55.9)	3 (37.5)	1 (100)	112 (54.6)	0.869 (0.758 to 0.995)	.03[a]
	DAS 28 ≤ 3.2 among patients with intervention, n (%)	119 (90.8)	35 (70.0)	5(50.0)	1 (50.0)	0	41 (66.1)	0.728 (0.604 to 0.877)	.04[a]

The Risk ratio and *P* value were calculated using a two-by-two contingency table.

[a] Chi-squared test

[b] Fisher's exact test

© 2023 Li C et al. *JAMA Network Open.*

eTable 7. Safety Data From Baseline to Month 6

	SSDM arm (n=1099)	Control arm (n=1098)
Patients with adverse events, n (%)	58 (5.3)	5 (0.5)
Events, total number, n (%)	74 (6.7)	5 (0.5)
Reported by rheumatologist, n (%)	2 (0.2)	2 (0.2)
Death, n (%)	2 (0.2)	2 (0.2)
Reported from SSDM[a], n (%)	72 (6.6)	3 (0.3)
Abnormal blood test results obtained from SSDM, n (%)	49 (4.5)	1 (0.1)
Liver and biliary disorders		
Hepatic enzymes increased[b], n (%)	7 (0.6)	1 (0.1)
Blood disorders		
Leukocytosis, n (%)	31 (2.8)	0
Leukopenia, n (%)	9 (0.8)	0
Thrombocytopenia, n (%)	2 (0.2)	0
Reported by patients (symptoms and signs), n (%)	23 (20.9)	2 (0.2)
Neurological disorders, n (%)	8 (0.7)	1 (0.1)
Cardiovascular disorders, n (%)	2 (0.2)	0
Skin and appendages disorders		
Dermatitis, n (%)	4 (0.4)	0
Alopecia, n (%)	1 (0.1)	1 (0.1)
Gastrointestinal disorders		
Mucosal ulceration, n (%)	2 (0.2)	0
Gastric function disorder, melaena, n (%)	5 (0.5)	0
Metabolic and nutritional disorders		
Hyperglycemia, n (%)	1 (0.1)	0

Data are n (%). SSDM = Smart System of Disease Management. [a] Reported from SSDM by patients retrospectively when they returned to the clinic at 6 months. [b] Alanine aminotransferase (ALT) or aspartate aminotransferase (AST) levels increased.

© 2023 Li C et al. *JAMA Network Open.*

eFigure. The Rate of Patients With DAS28 (CRP) ≤ 3.2 at Month 6

Sensitivity analyses of the primary endpoint. ITT, intent-to-treat. PP, per-protocol. IPCW, inverse probability of censoring weighted. SSDM, Smart System of Disease Management

	SSDM	Control	Group difference (95%CI)	*P* value	Risk ratio (95%CI)
Modified ITT analysis of multiple imputation	71.0%	64.5%	6.6% (2.7% to 10.4%)	.001	1.10 (1.04 to 1.17)
Worst case scenario	63.3%	52.6%	10.2% (6.2% to 14.1%)	<.001	1.19 (1.11 to 1.28)
PP	71.9%	65.1%	6.8% (2.7% to 10.9%)	.001	1.10 (1.04 to 1.17)
IPCW	71.7%	64.5%	7.2%(3.1% to 11.0%)	<.001	1.11 (1.05 to 1.18)

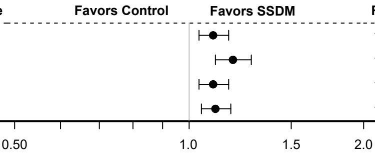

© 2023 Li C et al. *JAMA Network Open.*
