# Supplementary material for: Management of Rheumatoid Arthritis With a Digital Health Application: A Multicenter, Pragmatic Randomized Clinical Trial
Source: JAMA Netw Open. 2023 Apr 14;6(4):e238343. doi: 10.1001/jamanetworkopen.2023.8343 (PMC10105314; doi:10.1001/jamanetworkopen.2023.8343)
Supplement: Supplement 3. — Data Sharing Statement [file jamanetwopen-e238343-s003.pdf]

Data Sharing Statement

Li. Management of Rheumatoid Arthritis With a Digital Health Application: A Multicenter, Pragmatic Randomized Clinical Trial. *JAMA Netw Open*. Published April 14, 2023. doi:10.1001/jamanetworkopen.2023.8343

Data
Data available: Yes
Data types: Deidentified participant data
How to access data: murongster@163.com
When available: With publication

Supporting Documents
Document types: None

Additional Information
Who can access the data: Researchers whose proposed use of the data has been approved
Types of analyses: For analysis of results for approved research purposes (e.g. meta-analysis or systematic reviews)
Mechanisms of data availability: After approval and with a signed data agreement
Any additional restrictions: Additional Information of SSDM The proprietary of SSDM：Shanghai Gothic Internet Technology Co., Ltd How to access：Free to download via IOS and Android APP Stores (English version and Chinese version) Fee：Free to use The profit: Nobody profits from SSDM
